# NGF-Induced Cell Differentiation and Gene Activation Is Mediated by Integrative Nuclear FGFR1 Signaling (INFS)

**DOI:** 10.1371/journal.pone.0068931

**Published:** 2013-07-10

**Authors:** Yu-Wei Lee, Ewa K. Stachowiak, Barbara Birkaya, Christopher Terranova, Mariolina Capacchietti, Peter Claus, John M. Aletta, Michal K. Stachowiak

**Affiliations:** 1 Department of Pathology and Anatomical Sciences, State University of New York at Buffalo, Buffalo, New York, United States of America; 2 Western New York Stem Cells Culture and Analysis Center, Buffalo, New York, United States of America; 3 CH3 BioSystems LLC, New York State Center of Excellence in Bioinformatics & Life Sciences, Buffalo, New York, United States of America; 4 Institute of Neuroanatomy, Hannover Medical School, Hannover, Germany; 5 The Center for Systems Neuroscience, University of Veterinary Medicine Hannover, Hannover, Germany; University of Massachusetts Medical, United States of America

## Abstract

Nerve growth factor (NGF) is the founding member of the polypeptide neurotrophin family responsible for neuronal differentiation. To determine whether the effects of NGF rely upon novel Integrative Nuclear FGF Receptor-1 (FGFR1) Signaling (INFS) we utilized the PC12 clonal cell line, a long-standing benchmark model of sympathetic neuronal differentiation. We demonstrate that NGF increases expression of the *fgfr1* gene and promotes trafficking of FGFR1 protein from cytoplasm to nucleus by inhibiting FGFR1 nuclear export. Nuclear-targeted dominant negative FGFR1 antagonizes NGF-induced neurite outgrowth, *doublecortin (dcx)* expression and activation of the *tyrosine hydroxylase (th)* gene promoter, while active constitutive nuclear FGFR1 mimics the effects of NGF. NGF increases the expression of *dcx*, *th*, *βIII tubulin*, *nurr1 and nur77*, *fgfr1*and *fibroblast growth factor-2 (fgf-2)* genes, while enhancing binding of FGFR1and Nur77/Nurr1 to those genes. NGF activates transcription from isolated NurRE and NBRE motifs. Nuclear FGFR1 transduces NGF activation of the Nur dimer and raises basal activity of the Nur monomer. Cooperation of nuclear FGFR1 with Nur77/Nurr1 in NGF signaling expands the integrative functions of INFS to include NGF, the first discovered pluripotent neurotrophic factor.

## Introduction

Neuronal differentiation is fundamentally important for understanding normal human development as well as the implementation of new therapeutic interventions for neurological diseases. Development of the nervous system requires coordinated regulation of multi-gene programs by a plethora of extracellular and intracellular signals that facilitate the cell transition from the proliferative to differentiated state [1], [2]. NGF was the first of many ontogenetic signals identified for the development of the nervous system [3]. NGF is the founding member of the polypeptide neurotrophin family, activates transmembrane tyrosine kinase receptor TrkA [4] and is responsible for the survival and differentiation of sympathetic and dorsal root ganglion neurons, as well as other cells (neuronal and non-neuronal) in both the central nervous system and the periphery [5]. The PC12 rat adrenal pheochromocytoma cell line is an experimental model system used extensively to study neuronal differentiation and has revealed many aspects of the NGF mechanism of action [6], [7]. NGF induces biochemical, electrophysiological and morphological (neurite outgrowth) changes in PC12 cells that recapitulate many features characteristic of differentiated sympathetic neurons [8], [9]. Studies on PC12 cells have enabled a quantitative picture of proximal NGF signaling events based on a uniform homogeneous population of cells [10].

Important effectors of the NGF mechanism include the cytoplasmic/nuclear kinases, including ribosomal S6 kinase 1 (RSK1) [11], and Nur nuclear orphan receptors [12]. NGF targets the RSK family of cellular kinases and endogenous RSK1 is sufficient for PC-12 differentiation [11], [13]. Among the nuclear sequence specific transcription factors (ssTF) that transduce NGF signals, Nur77, also referred to as NGFI-B, is one of the immediate early genes originally identified by rapid activation in PC12 cells [12]. Nur77,together with related proteins Nurr1 and NOR-1, comprise a group of nuclear orphan receptors that are devoid of a ligand-binding domain and function as ssTF for the expression of various genes within multiple signaling pathways. Nur77, Nurr1 and NOR-1 are expressed in numerous tissues, including the brain, and play roles in cell proliferation, differentiation, and apoptosis [14], [15], [16], [17], [18], [19], [20]. Nurs integrate diverse developmental neuronogenic signals including those generated by NGF [12], cyclic AMP(cAMP) [21] and retinoic acid (RA) and participate in important pathways for PC12 differentiation [12], [21].

Recent studies have shown that both RSK [22], [23] and Nur [24], [25] are involved in the universal Integrative Nuclear FGFR1 Signaling (INFS) gene regulating mechanism [2], [23], [26], [27], [28], [29], [30]. INFS influences gene activities and controls cell development utilizing a direct nuclear action of FGFR1 initiated by diverse neurogenic factors, including RA, cAMP and BMP7. Studies revealed atypical structural features of the FGFR1 transmembrane domain (TMD) and novel interactive features of FGFR1 which allow the newly synthesized 90 kDa protein to be released from preGolgi membranes and translocate into the cell nucleus along with the Nuclear Localization Signal (NLS)-containing FGF-2 ligand [23], [31], [32], [33]. FGFR1 is transported to the nucleus by NLS binding importin-β [34]. Nuclear (n)FGFR1 is a highly mobile chromatin protein [35] which binds and activates CREB binding protein (CBP) and Ribosomal S6 kinase-1 (RSK1). FGFR1 forms complexes with retinoid and Nur receptors and “feeds forward” developmental signals directly to CBP and RSK1. The coupled activation of CBP and RSK1 by nuclear FGFR1, and cascade signal transduction to ssTF, enable coordinated gene regulation and cell differentiation and has been referred to as “feed-forward-and-gate” signaling [23], [27].

Among the genes involved in neuronal differentiation, only a few have been studied in relation to regulatory control by nuclear FGFR1, Nurs and RA receptors [30]. Nuclear FGFR1 increases the expression of *th, neurofilament, neuronal enolase* and *fgf-2* and chromatin immunoprecipitation (ChIP) experiments showed nuclear FGFR1, together with CBP and other DNA binding proteins, associates within the promoters of the *th* and *fgf-2* genes [23], [24], [25].

Yeast two-hybrid and coimmunopreciptation assays revealed that the FGFR1 tyrosine kinase domain binds directly to RSK1 N-terminal kinase [22], [23]. RSK1 binding promotes FGFR1 release from pre-Golgi to cytosol, increases the mobile population cytosolic of FGFR1 and facilitates nuclear accumulation of FGFR1 [33]. In the cell nucleus interaction of FGFR1 with RSK1 restricts the FGFR1 intra nuclear mobility and promotes RSK1 activation of CREB [32], [33]. In addition our recent studies showed that FGFR1 forms nuclear complexes with both the Nurr1 and Nur77 proteins.

Given that RSK and Nur77 are fundamentally involved in nuclear signaling through both NGF and INFS, the possibility that NGF may utilize the INFS mechanism for neurodevelopmental and gene-activating functions has now been examined. We report that NGF promotes FGFR1 cytoplasmic-nuclear trafficking, in part, by inhibiting FGFR1 nuclear export. Furthermore, nuclear FGFR1 is essential for NGF-induced differentiation and transcriptional programming ofPC12 cells.FGFR1 binds to Nur-targeted regions of NGF-activated genes and augments NGF activation of ligand-independent function of Nur77/Nurr1. The present study provides a new perspective on the diverse actions of NGF (e.g. gene expression, neurite outgrowth) which requires the neurodevelopmental INFS mechanism.

## Materials and Methods

### Plasmids

Plasmids expressing wild type FGFR1, constitutive nuclear FGFR1(SP−/NLS), with the signal peptide replaced with an NLS which accumulates in the nucleus in a ligand-independent manner, tyrosine kinase-deleted dominant negative nuclear/cytoplasmic FGFR1(TK-)-and nuclear FGFR1(SP−/NLS)(TK-) were described in [26], [36]. Plasmid FGFR1-EGFP was described in Fang et al., 2005 [23] and Dunham et al., 2009 [32]. The reporter plasmid *th*-luciferase (Luc) containing −425/+25 bp fragment of bovine *th* promoter was previously described [37]. Nurr1 expressing pCAGGS-Nurr1-FLAG plasmid was generated from pCAGGS-empty provided by Dr. Hitoshi Niwa, RIKEN Center for Developmental Biology, Japan [38], which comprised the CAG-promoter composed of CMV immediate early enhancer and chicken β-actin promoter. Plasmids NurRE3-Luc containing three Nur response elements and NBRE3-Luc containing three NGF binding response elements in the minimal POMC gene promoter (−34/+63) and Nur77 expressing pCMX vector were gifts from Dr. Jacques Drouin (Institut de RecherchesCliniques de Montrèal) [39], [40].The reference reporter plasmid (pGL4.70 [hRluc] promoterless) was purchased from Promega Corp. (Madison, WI).

### Antibodies

Monoclonal mouse αFGFR1 (N-terminal)(ab823) and polyclonal rabbit αFGFR1 (C-terminal, sc-121) were purchased from Abcam (Cambridge, MA) and from Santa Cruz Biotechnology (Santa Cruz, CA), respectively. The N-terminal αFGFR1 mcAb6 was described in Hanneken et al. [41]. αNurr1/77 (sc-990) and αGADPH (sc-137179) was purchased from Santa Cruz Biotechnology (Santa Cruz, CA). Rabbit αMatrin-3 Ab (A300-591A) was from Bethyl Laboratories (Montgomery, TX). Rat αBrdUAb (MCA2060) was purchased from AbDSerotec (Raleigh, NC). Rabbit IgG (X0903) was from Dako (Carpentaria, CA). Specificity of immunostaining was ascertained with control reactions in which the primary Ab was omitted or replaced with pre-immune serum or by neutralizing the antibody with cognate peptide [2], [24], [25], [42]. In addition the specificity of sc-121 and AbcamαFGFR1 was demonstrated independently by Chioni and Grose 2012 [43].

### Cell Culture, Transfection, Drugs and Neurite Outgrowth and Neurite Regeneration

PC12 cells were drawn from cryopreserved isolates of the original cell line created by Greene and Tischler [6] and cultured in RPMI1640 medium supplemented with 10% donor horse serum, 5% fetal bovine serum, 25 U/ml penicillin and 25 micrograms/ml streptomycin. During NGF (50 ng/ml) treatment, the fetal bovine serum was removed and the donor horse serum was reduced to 1%. LMB was added at a concentration of 100 ng/ml in 0.1%ethanol. Control cultures were maintained in 0.1% ethanol.

Human neuroblastoma cell line BE(2)C (ATCC, Manassas, VA) was cultured in Dulbecco modified Eagle medium/Nutrient Mixture F-12 (DMEM/F-12, Gibco) supplemented with 10% (v/v) fetal bovine serum, 100 U/ml penicillin/0.1 mg/ml streptomycin and non-essential amino acid (Gibco). All cells were cultivated in a humidified atmosphere at 37°C and 5% CO2. Cell transfections were performed using Lipofectamine 2000 (Invitrogen). LMB was purchased from LC Laboratories (MA, 01801).

The neurite lengths of PC12 cells transfected with an EGFP expression plasmid were imaged under 20×or 40×magnification as previously described [1], [23], [24]. Cell bodies and extensions of the fluorescent cells were outlined using the ImageJ freehand tracing tool or AxioVision Rel. 4.8. Processes greater than 1 cell diameter in length were scored. Cells with shorter protrusion were scored as having neurite length equal to zero. The overall statistical differences identified by One-Way ANOVA were analyzed further by LSD’s post-hoc test.

An assay for neurite regeneration [44] was employed to determine the effects of nuclear FGFR1 and the dominant negative form of FGFR1 on neurite extension. PC12 cells were primed in 50 ng/ml NGF in culture. After 2 weeks of NGF treatment, cells were transfected (nuclear FGFR1 or dominant negative FGFR1 and EGFP to mark successfully transfected cells) in serum-free and NGF-free medium containing 2 mM insulin. After transfection, the cells were returned to NGF-containing medium. Thirty-six hours later, NGF was removed from the cells by extensive washing in RPMI +1% horse serum. The neurites were sheared from the cell bodies by trituration with a Pasteur pipet followed by further washes with RPMI +1% horse serum through 3 cycles of centrifugation and cell re-suspension prior to re-plating ± NGF. The percentage of EGFP-expressing neurite-bearing cells relative to the total number of EGFP-expressing cells observed was scored by use of a fluorescence microscope.

### Dual Luciferase Assays

Transcription assays were performed with the dual luciferase reporter system (Promega Corp., Madison, WI)as described in Yu-Wei Lee et al., 2012, Baron et al., 2012 [24], [25]. Luminescence measurement was performed on BioTek Plate Reader(Winooski, VT). The data were calculated as the ratio of firefly to Renilla luciferase activity or normalized by protein concentration and transfection rate evaluated by co-transfection of EGFP. Experiments were repeated 2 to 4 times and each was performed in quadruplicate.

### Cell Fractionation & Western Blotting

Cells were fractionated as described in [26] and [2] and equal amounts of protein from cytoplasmic or nuclear fractions were loaded and separated on SDS-7.5% polyacrylamide gel and transferred to PVDF (Millipore). The purity of fractions were verified in previous studies [2], [42]. Blots were probed with the appropriate antibodies and the immune complexes revealed by chemiluminescence using SuperSignalFemto Maximum Sensitivity Substrate (Pierce, Rockford, IL) and Fuji chemiluminescence imager. Equal protein content in individual lanes was verified by immunoblotting for GADPH (a predominantly cytoplasmic protein) andMatrin-3 (a predominantly nuclear protein) [24], [45], [46].

### Fluorescence Loss In Photobleaching (FLIP)

FLIP of transiently transfected FGFR1-EGFP [32], [33] was performed on Zeiss 510 Meta confocal laser scanning microscope with an incubation chamber (37°C and 5% CO2, PeConGmbH, Erbach, Germany). The transfected recombinant proteins were expressed at the levels comparable to endogenous proteins [32], [33]. The intensities of FGFR1-EGFP fluorescence in individual transfected cells were similar and cells were randomly selected for FLIP measurements. Bleaching and imaging were performed in 35 mm glass bottom dishes (MatTek Corp., Ashland, MA) using Zeiss 510 Meta confocal laser scanning microscope with an incubation chamber and oil immersion objective (63×, 1.4 NA), a zoom magnification (3-fold), the 488-nm argon laser line for GFP. Intensity of fluorescence in individual transfected cells was similar, and cells were randomly selected for the FLIP measurements.

For FLIP recording in the cytoplasm, the fluorescence intensity in the cytoplasmic regions of interest were averaged from three images with 5 sec interval before photo-bleaching. At least two cytoplasmic regions of interest, excluding the endoplasmic reticulum, were chosen in each cell. The laser output for FLIP-bleaching was set to 100%. Approximately 1/3 of the nuclear area was bleached followed by two scans with 2.5 sec intervals (image acquisition). The bleaching/scanning cycle was repeated 50 times.

The image series on Fig. 2 illustrate the fluorescence intensity before bleaching (0 s) and after consecutive bleaching periods. Acquired images from a minimum of 11 cells/conditions were collected and analyzed using the standard microscope software. Image scanning resulted in a loss of fluorescence in all cells including non-photobleached cells. To compensate for this loss, the loss of fluorescence in photobleached cells was reduced by an amount equivalent to the loss measured in non-photobleached cells. The kinetic constant, k, and the mobile fraction for a FLIP experiment were calculated with FCalc software by fitting an exponential curve to the corrected data using a least square fit [33]. The exact FLIP formulas used for one function fit are: 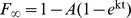; A is the mobile fraction and k is the kinetic constant. In addition to these parameters the half time, t_1/2_, of the reaction is given. The value of the half time, t_1/2_ recovery was calculated with the following formula, 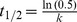. t_1/2_ is the time point when the signal has reached 50% of the final value. SigmaPlot and SPSS were used for plotting of the data and statistical analysis. ANOVA tests were applied to analyze differences among recovery half-times (t_1/2_), populations of mobile FGFR1-EGFP and the effects of drugs.

For the cytoplasmic bleaching a randomly chosen region of cytoplasm (outside the Golgi) was bleached and the fluorescence intensity measured in the cytoplasmic and nuclear regions of interests as described above for the nuclear bleaching.

### Immunocytochemistry

Cells were fixed with 4% paraformaldehyde and permeabilized with 1% Triton X-100. Appropriate primary and secondary antibodies (described in the legends of figures) as well as DAPI were applied for immunostaining [23], [42]. Immunostaining was observed using either Zeiss Axioimager fluorescence microscope with a 20x or 40x oil objective or a Zeiss 510 Meta confocal laser scanning microscopes (Thornwood, NY) with an oil immersion objective (63×, 1.4 NA), the 488-nm argon laser line, the 561-nm DPSS laser line, 633-nm HeNe laser line and Chameleon laser line (Coherent Inc.) for DAPI. The specificity of FGFR1 immunostaining was demonstrated as previously [2], [23], [32], [33], [47] by several observations: Staining was not observed when the primary antibody was omitted or replaced with preimmune serum (not shown). Similar nuclear-cytoplasmic FGFR1 localization and FGFR1 DNA binding were observed by using three antibodies targeting different FGFR1 epitopes and by detection of transfected FGFR1-EGFP and FGFR1-Flag using native fluorescence and αFlag [24]. The presence and changes in the levels of nuclear FGFR1 immunoreactivity were confirmed by Western blot analysis of FGFR1 in subcellular fractions.

### Quantitative Immunocytochemistry of Doublecortin and 0.1 mM 5′-flurouridine (FU) Labeled RNA

PC12 cells were transfected with EGFP expressing plasmid and immunostained with polyclonal rabbit DCX antibody (Santa Cruz) plus Cy3 conjugated secondary antibody. Samples were prepared side-by-side at the same time using the same reagents/buffer. Cells were imaged using Zeiss 510 Meta confocal laser scanning microscopes (Thornwood, NY) at the 0.35 µm z-section interval with an oil immersion objective (63×, 1.4 NA). The laser intensity (50% of total output, 8% laser intensity for FITC and 6% for Cy3), zoom, offset, gain, pinholes and scanning time were held constant within the linear range to quantitatively compare the samples. Images were compressed using ImageJ software into a single z-projection/stack which was the sum of the optical sections in the stacks. The intensity of DCX-IR pixels in individual outlined EGFP+ cells was measured using ImageJ as previously described [33]. The same sufficiently low laser intensity was applied to all cell images to prevent saturation of pixels in z-projection/stack.

PC12 cells were incubated with 0.1 mM 5′-flurouridine (FU) for 25 minutes to label newly synthesized RNA. The FU-RNA was detected using polyclonal rat anti-BrdU plus Alexa 568 conjugated rabbit anti-mouse secondary antibody [24]. Whole individual nuclei were imaged using Zeiss 510 confocal microscope at the 0.35 µm z-section interval with the constant laser intensity (50% of total output, 6% laser intensity for both FITC and Cy3). The same microscope parameters and z-projection/stack were utilized for DCX Immunocytochemistry. The FU-IR pixel intensity was measured in the nuclei of EGFP-transfected green cells using imageJ.

### mRNA Level Determination using Quantitative PCR

Total RNA was isolated from 10 mm plates of PC12 cell cultures using RNAeasy mini kit (Qiagen, Valencia, CA). cDNA synthesis was carried out using 2 µg RNA and the iScriptcDNA Synthesis Kit (Bio-Rad, Hercules CA). One twentieth of the synthesized cDNA was used as the template for real-time PCR. 25 µl real time PCR reactions were performed on the BioRadMyiQ Cycler with iQ SYBR Green Supermix (Bio-Rad,Hercules, CA). RT-qPCR using the amplification cycles: initial denaturation for 8 min at 95°C, followed by 35×cycle 2 (denaturation for 15 sec at 95°C and annealing for 1 min at 60°C). Melt curve data collection was enabled by decreasing the set point temperature after cycle 2 by 0.5°C. The specificity of amplicons was confirmed by generating the melt curve profile of all amplified products. Gene expression was quantified as described [48]. Primers are listed in Table S1.

### In vitro and in vivo ChIP Assays

The assay was performed in vitro as described earlier in [24]. Cells grown on a 10 mm plate were cross-linked with 1% formaldehyde (Sigma, St Louis, MO) at 37°C for 10 minutes, rinsed twice with cold phosphate-buffered saline and harvested in phosphate-buffered saline with protease inhibitors by 5 min centrifugation at 2,000 g. ChIP was performed according manufacturer’s instructions (Millipore, Temecula, CA). Genomic DNA was precipitated with ethanol and after treatment with RNase A and proteinase K, purified using Qiagen PCR purification kit. qPCR was then performed on the immunoprecipitated genomic DNA with primers for the response element containing regions of listed genes (Table 1) and the control *cyclophilinA* gene. All primers amplifying these promoter regions are shown in Table S2.

**Table 1:** Potential Nur-binding sites in tested genes.

Gene	Site	Type	Location	Sequence
TH	NBRE	Consensus site	Intron1	TGACCTTT
TH	NurRE-like site	Potential site	Promoter	ATACCA
DCX	NBRE	Consensus site	Intron1	AAAGGTCA
DCX	NurRE-DR2 site	Consensus site	Intron1	TGAGTATxxTGAGCAT
FGF2	NBRE	Consensus site	Intron1	TGACCTTT
FGF2	NurRE-DR2 site	Consensus site	Intron1	TGACATxxxxxxGATATTTxxTGAGTATA
Nur77	NurRE-like site	Potential site	Promoter	TGGTATTT
Nurr1	NurRE-like site	Potential site	Intron1	TGGCATAT
BIII-Tubulin	NBRE-like site	Potential site	Promoter	TGACCT
BIII-Tubulin	NurRE-like site	Potential site	Intron1	TGAGTAT

The assay was performed in vivo as described in Baron et al., 2012 [25]. Rats were killed by CO_2_ asphyxiation followed by decapitation using protocol approved by the State University of New York at Buffalo Institutional Animal care and Use Committee (IACUC). Brains were quickly removed and dissected on ice into: brain cortex, cerebellum, olfactory bulbs and ventral midbrain (substantia nigra region). Tissues were minced and incubated in cross-linking solution: cold phosphate-buffered saline with 1% (w/v) formaldehyde (Sigma, St Louis, MO) at room temperature for 15 minutes, rinsed twice with cold phosphate-buffered saline and sonicated in phosphate-buffered saline with protease inhibitors. Sonicated fragments were centrifuged at 14,000 g, 10 min at 4°C. ChIP was performed in samples containing equal amount of genomic DNA using polyclonal antibodies: rabbit FGFR1 (ab10646, Abcam, Cambridge, MA), Nur77/Nurr1 (sc-5568, Santa Cruz, CA) or control rabbit IgG provided by the MAGnify Chromatin Immunoprecipitation System and validated in [24], [25]. The FGFR1 antibodies were also ChiP validated independently by Chioni and Grose, 2012 [43]. DNA was purified according manufacturer’s instructions (Invitrogen, Grand Island, NY). Sample PCR was then performed on the immunoprecipitated genomic DNA with primers for the response element containing regions of the *th* and *dcx* genes.

### qPCR Analysis of Chip

qPCR was used to determine relative amount of specific loci in IP, Input, and control IgG(Pre-immune) samples. qPCR was performed using iQ SYBR Green Supermix (Bio-Rad, Hercules, CA) on a Bio-Rad iCycler. Three to five microliters of CHIP DNA and a 1∶10 dilution of input DNA was used in duplicate reactions. Data are expressed as IP/input where:
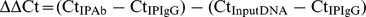



The PCR assays were performed at least three times and the results combined and shown as relative change means +/− SEM.

### Statistical Tests

SigmaPlot 12.0 and SPSS were used for plotting the data and statistical analysis. For all data, the overall statistical significance was determined by ANOVA followed by a post-hoc LSD analysis of differences between specific groups. Interactions between variable factors were determined using Two-Way ANOVA.

## Results

### NGF Stimulates FGFR1 Nuclear Trafficking

**Figure 1:**
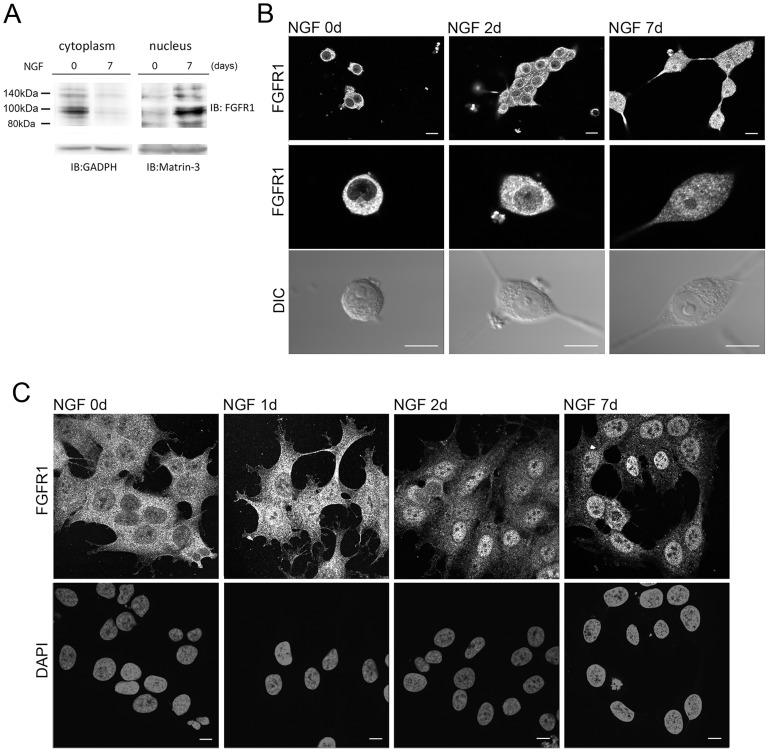
NGF induces FGFR1 nuclear accumulation in PC12 and neuroblastoma cells. (**A**) PC12 cells were maintained in RPMI1640 with 1% horse serum in the presence or absence of NGF (50 ng/ml) for 7 days. The cytoplasmic and nuclear proteins were prepared for electrophoresis (45 µg protein/lane) and immunoblotting with monoclonal N-terminal FGFR1 antibody (Abcam). Cytoplasmic and nuclear immunoreactive FGFR1 protein bands of 140, 110, 100 and 90 kDa correspond to different degrees of FGFR1 glycosylation [27]. Normalization to GADPH and matrin-3 verifies cytoplasmic depletion and nuclear accumulation of FGFR1, respectively. (**B**) PC12 cells were treated with 50 ng/ml NGF for the durations indicated or maintained without NGF. The effect of NGF on FGFR1 expression and nuclear accumulation is illustrated by immunostaining with monoclonal N-terminal FGFR1 antibody plus goat-anti mouse Alexa488. (**C**) Human neuroblastoma cells were incubated with or without 100 ng/ml NGF for 1, 2 or 7 days and immunolabeled with N-terminal FGFR1 antibody plus goat-anti mouse Alexa488. Nuclear DNA was stained with DAPI. Nuclear accumulation of FGFR1 is observed after NGF treatment. Bar length - 10 µm (B and C).

The principal action of NGF on neuronal progenitor cells or PC12 cells is the induction of neuronal differentiation characterized by time-dependent morphological, biochemical and physiological changes [8], [44]. To determine whether NGF action involves the INFS mechanism we first analyzed the expression and subcellular localization of FGFR1 in PC12 cells. In control non-treated PC12cells, the 100 and 140 kDa forms of FGFR1 are expressed in the cytoplasm (Fig. 1A). These forms of the receptor are known to represent different degrees of receptor glycosylation [49]. Both forms are depleted in the cytoplasm after 7 days of NGF and a concomitant increase in nuclear 100 and 140 kDa FGFR1 is observed. The complementary changes in cytoplasmic and nuclear FGFR1 levels illustrate a lack of cross-contamination in isolated fractions [22], [24]. In addition, NGF increases nuclear 80–90 kDa FGFR1, which are the precursors of the hyper-glycosylated forms [33]. Consistent with these biochemical results, in non-treated PC12 cells, FGFR1 immnunoreactivity (IR) is primarily cytoplasmic (Fig. 1B). After two days of NGF treatment we observed intense nuclear FGFR1-IR foci with a further increase in FGFR1-IR after 7 days of treatment. Nuclear accumulation of FGFR1 was detected using monoclonal N-terminal FGFR1 Ab from Abcam (Fig. 1A, B) as well the polyclonal C-terminal FGFR1 Ab from Santa Cruz (Fig. S1A, supplementary material) and the N-terminal FGFR1mcAB6 (Fig. S1B,C supplementary material), giving further support to the observations of nuclear accumulation of full length FGFR1 [2].Importantly, the nuclear accumulation of truncated FGFR1 (approximately 50 kDa) was demonstrated in breast cancer cells [43]. In addition, our confocal analyses show small increases of FGFR1-IR nuclear foci after only one hour of NGF treatment (Fig. S1B supplementary material), indicating an early activation of nuclear FGFR1 trafficking by NGF. This early FGFR1 nuclear accumulation is augmented by Leptomycin B (LMB) (Fig. S1C supplementary material), which blocks CRM1-dependent nuclear export of proteins [50], [51]. Treatment with NGF also induces a similar nuclear accumulation of FGFR1 in human neuroblastoma cells (Fig. 1C). We conclude that the nuclear accumulation of FGFR1 represents a common response during NGF-induced cellular differentiation.

**Figure 2:**
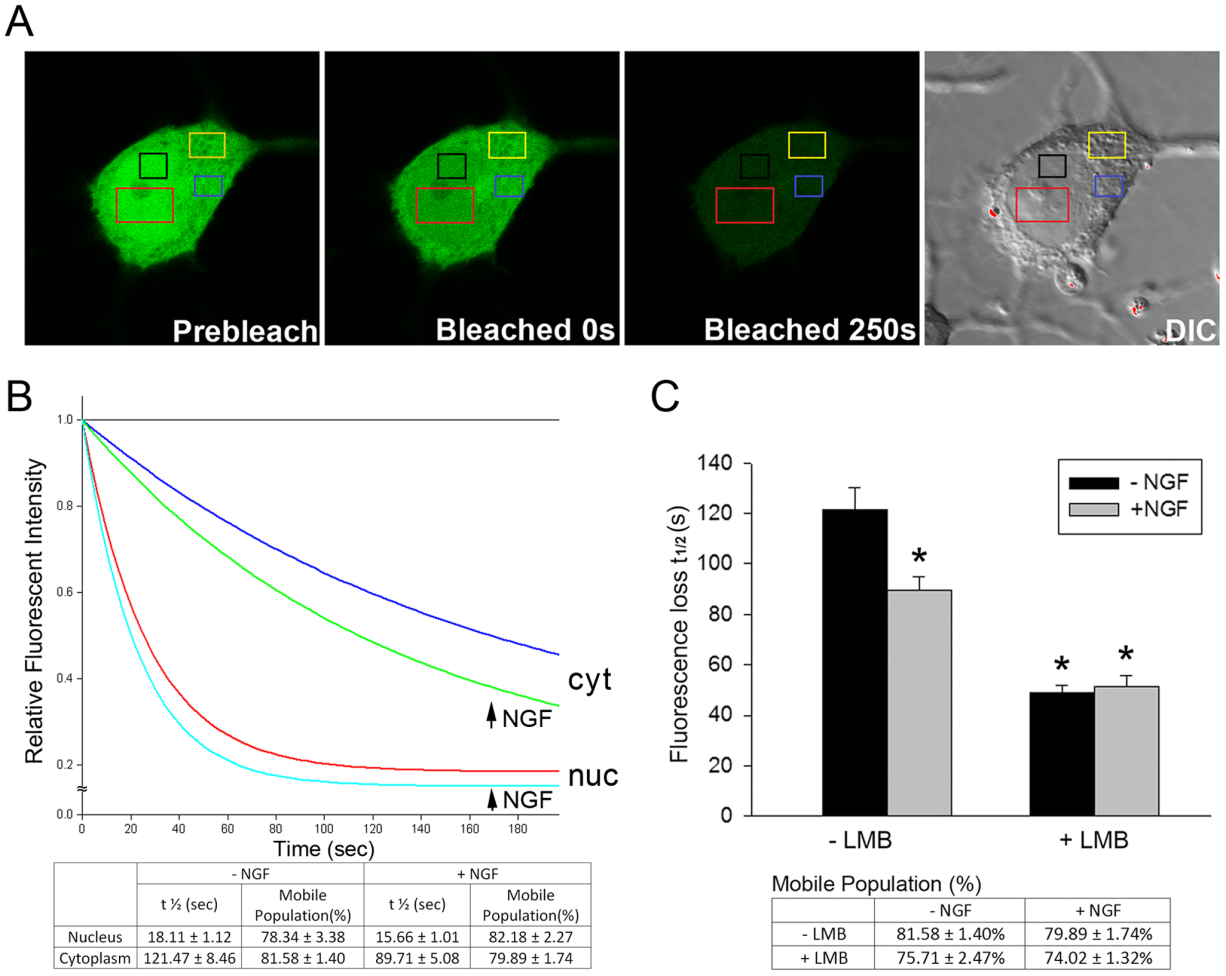
NGF accelerates nuclear trafficking of FGFR1 by reducing FGFR1 nuclear export. (**A**) FGFR1-EGFP was transfected into PC12 cells. Twenty-four hours after transfection, cultures were treated with 50 ng/ml NGF and LMB (100 ng/ml in 0.1% v/v ethanol) or ethanol alone (0.1% v/v ethanol) for an additional 48 h and during subsequent imaging. Examples of FGFR1-EGFP expressing cells before and after photo-bleaching are shown. DIC image indicates the nuclear and cytoplasmic regions. About 1/3 nuclear area of PC12 cell was bleached by high intensity laser and 2–3 regions of interest (ROI) intensity were measured. (**B**) Single-exponential analysis of FGFR1-EGFP FLIP regression in cytoplasm and nucleus showed that the diffusion rate of FGFR1-EGFP is affected by NGF treatment in live cells. Individual curves represent means of at least 23 cells. NGF significantly increases the FGFR1-EGFP exchange between nucleus and cytoplasm (half-time decreases) without affecting the FGFR1-EGFP mobile population. (**C**) Single-exponential analysis of FGFR1-EGFP FLIP regression in the cytoplasm shows that NGF facilitates FGFR1-EGFP trafficking between the cytoplasm and nucleus (half-time decreases from 121.5 sec to 89.7 sec; One-way AVOVA, LSD*p<0.001 different to -LMB/−NGF). LMB (100 ng/ml) alone markedly reduces the FGFR1-EGFP half-time (*p<0.001 different to -LMB/−NGF). However, no additive effect of NGF and LMB was observed (p = 0.76), indicating that NGF accelerates FGFR1 nuclear accumulation by reducing nuclear export.

To verify further if NGF facilitates the dynamic FGFR1 exchange between cytoplasmic and nuclear compartments we performed Fluorescence Loss Induced by Photo-bleaching (FLIP) on PC12 cells transfected with FGFR1-EGFP. FGFR1-EGFP expression in both the cytoplasmic and nuclear compartments is similar to that of endogenous FGFR1 and mimics the actions of non-fused FGFR1 [1], [32]. In FLIP experiments, repeated photo-bleaching of FGFR1-EGFP in a small defined nuclear region results in a rapid (t_1/2_ = 18 sec) loss of FGFR1-EGFP fluorescence within the entire nucleus as well as a delayed (t_1/2_ = 124 sec) loss in the cytoplasm (Fig. 2A,B). These experiments demonstrate that (i) nuclear and cytoplasmic FGFR1 are indeed present in distinct subcellular-kinetic compartments and (ii) nuclear FGFR1 remains in equilibrium with the cytosolic receptor. LMB significantly (p<0.001) accelerated the decline of FGFR1-EGFP cytoplasmic fluorescence during nuclear bleaching, confirming that FGFR1 nuclear import is counteracted by ongoing nuclear export. Importantly, in NGF-stimulated cells, depletion of cytoplasmic FGFR1-EGFP after nuclear photo-bleaching was accelerated markedly, indicating that cytosolic to nuclear trafficking is increased (Fig. 2C). This NGF effect could reflect acceleration of FGFR1 nuclear import or reduced nuclear to cytoplasmic export. In LMB treated cells, NGF treatment failed to further accelerate the loss of cytoplasmic fluorescence induced by nuclear photo-bleaching. Thus, one mechanism through which NGF promotes nuclear accumulation of FGFR1 is a reduction of FGFR1 nuclear export, i.e., “arresting” the receptor in the nucleus.

During nuclear bleaching, regions of the cytoplasm immediately above or below the nucleus could become inadvertently bleached contributing to the decrease in fluorescence intensity in other investigated cytoplasmic ROI. To verify the FGFR1-EGFP exchange between the cytoplasmic and nuclear compartments and the effects of NGF on FGFR1-EGFP nuclear export we performed reverse FLIP experiments. A randomly chosen region of the cytoplasm (outside Golgi) was bleached and the losses of FGFR1-EGFP fluorescence were recorded in other cytoplasmic regions as well as the cell nucleus. The loss of FGFR1-EGFP fluorescence was approximately 5-times more rapid in the cytoplasmic ROI than in the nucleus, thus confirming the rate-limiting FGFR1-EGFP exchange between these two compartments (Fig. S2, supplementary material). Importantly, NGF treatment markedly slowed down the loss of nuclear FGFR1-EGFP fluorescence. This observation confirms the NGF-induced nuclear “arrest” of FGFR1.

### Nuclear FGFR1 is Essential for NGF Induced Neurite Outgrowth and Activation of the *th* Gene Promoter

Earlier studies from several laboratories have provided in depth characterization of NGF-induced PC12 neuronal-like differentiation i.e., an outgrowth of neurites with growth cone-like endings accompanied by an up-regulation of the neurotransmitter biosynthetic enzyme, TH, neuronal β-III Tubulin [52], MAP-2 [53], Neurofilament L [54], [55], NMDAR1 protein [56] and nicotinic acetylcholine receptor currents [57]. Other studies have also indicated that NGF can evoke neuron-specific voltage-dependent K+ and Na+ currents [58], [59].

**Figure 3:**
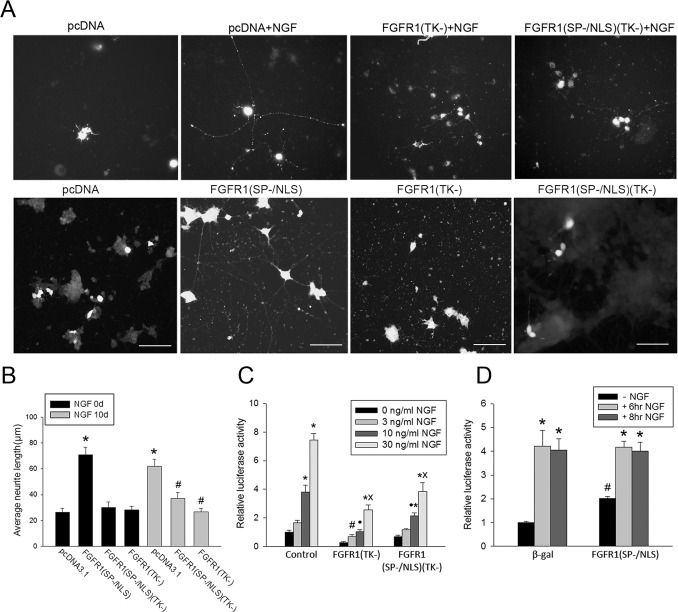
Nuclear FGFR1 mediates NGF induced neurite outgrowth and activation of the *th* gene promoter. (**A**) PC12 cells were transfected with two plasmids, one expressing recombinant FGFR1 or control pcDNA3.1 and the second expressing EGFP. EGFP diffuses throughout the cell permitting visualization of the entire neuritic network. More than 90% of cells co-expressed transfected plasmids as reported in previous studies [29], [73]. Twenty-four hours after transfection cultures were switched to 1% horse serum medium with or without (control) 50 ng/ml NGF for an additional 10 days, after which the cells were imaged using fluorescent microscopy. Bar length - 100 µm. (**B**) The longest process in an individual transfected cell was measured using ImageJ [24].* mark comparison to pCDNA3.1 (-NGF) and # to pCNA3.1 (+NGF). Transfection of constitutive nuclear FGFR1(SP−/NLS) increased neurite outgrowth approximately 3-fold (*p<0.001, One-Way ANOVA, LSD). A similar increase was induced by NGF treatment of pcDNA3.1 transfected cells (*p<0.001). Cells transfected with dominant negative FGFR1(SP−/NLS)(TK-) or FGFR1(TK-) display no significant changes in average neurite length in the absence or presence of NGF. (**C**) PC12 cells were transfected with a *th-* Luciferase reported plasmid [37] and dominant negative FGFR1(TK-), FGFR1(SP−/NLS)(TK-) or control pcDNA3.1(-). 24 hours after transfection cells were treated for an additional 24 hours with indicated concentrations of NGF. The *th*-Luc expression in the presence of NGF was significantly reduced by both FGFR1(TK-) and FGFR1(SP−/NLS)(TK-). Dominant negative FGFR1 constructs had no significant effect on basal promoter activity. One-Way ANOVA, LSD: * (p<0.01) - mark comparison to (-NGF) within individual plasmid transfection groups; **#**(p<0.05) - comparison to pCDNA3.1+3 ng/ml NGF; •(p<0.01) - comparison to pcDNA3.1+10 ng/ml NGF; and **x** (p<0.001) - comparison to pCNA3.1+30 ng/ml NGF. Two-Way ANOVA shows interactions (p<0.001) between NGF and plasmid constructs (control, FGFR1(TK-) or FGFR1(SP−/NLS)(TK-)). (**D**) PC12 cells were transfected with *th*-Luc and control pcDNA3.1(-) or pcDNA3.1 expressing an active constitutive nuclear FGFR1(SP−/NLS). 24 hours after transfection cells were treated for an additional 6 or 8 hours with NGF. FGFR1(SP−/NLS) increases *th* promoter activity 2-fold in the absence of NGF but shows no additive stimulation in the presence of NGF. One-Way ANOVA, LSD: * (p<0.001) - comparison to (-NGF) within individual plasmid transfection groups; **#**(p<0.05) - comparison to pCDNA3.1 (- NGF).

**Figure 4:**
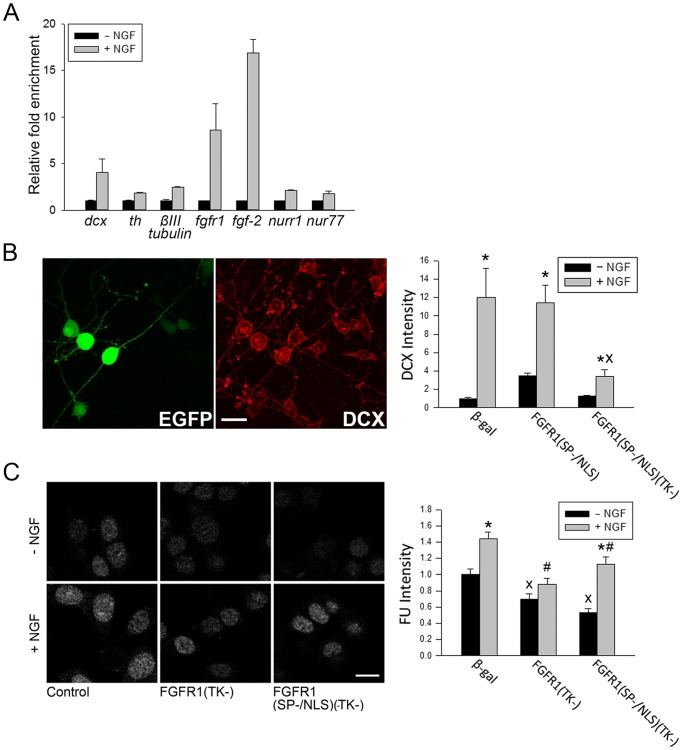
Gene activation and global RNA synthesis by NGF. (**A**)PC12 cells were incubated with NGF (50 ng/ml) or without NGF for 4 hours. mRNA levels were measured using RT-qPCR relative to the house keeping gene *cyclophilin A* mRNA. Representative experiment shows NGF-induced up-regulation of *fgfr1, fgf2, dcx, th, βIII-tubulin, nurr1* and *nur77* mRNAs. Similar results were observed in three experiments. (**B**) PC12 cells were transfected with recombinant FGFR1 or control β-gal and with EGFP to mark transfected cells. Images show examples of EGFP fluorescence (left) and DCX-IR (right) in the same β-gal transfected, NGF treated cells. Bars represent average DCX-IR intensity (±SEM) in the nuclei of least 12 EGFP+ cells measured using imageJ. Treatment with NGF for 7 days leads to a gradual outgrowth of neurites with intensified DCX-IR. Cells transfected with FGFR1(SP−/NLS)(TK-) and treated with NGF display significantly less DCX-IR (One-Way ANOVA, LSD *p<0.05 different to –NGF within individual plasmid groups; ^X^p<0.001 different from β-gal+ NGF. Two-Way ANOVA (p<0.001) shows an interaction between transfected plasmid DNA and NGF. Bar length - 20 µm. (**C**) PC12 cells were transfected with FGFR1(TK-), FGFR1(SP−/NLS)(TK-) or β-gal. After 24 hr, cells were incubated for an additional 24 hours with or without NGF, and subsequently with 0.1 mM 5′-flurouridine (FU) for 25 minutes to label newly synthesized RNA. FU-RNA was detected using anti-BrdU and confocal microscopy. Bars represent average (±SEM) intensity of nuclear 5′-FU-IR in at least 19 EGFP expressing cells. NGF significantly increased FU-IR intensity in PC12 cells. Dominant negative FGFR1 significantly decreased the FU-IR intensity in NGF treated and non-treated cells. One-Way ANOVA, LSD: *p<0.05 difference between – NGF and+NGF within individual plasmid groups; ^X^p<0.05 difference from β-gal -NGF; #p<0.05 difference from β-gal +NGF. Two-Way ANOVA (p = 0.057) suggests a potential interaction between transfected plasmid DNA and NGF. Bar length - 10 µm.

In the present work we find that NGF-induced nuclear accumulation of FGFR1 is accompanied by exit from the cell cycle (not shown), an acquisition of neuronal morphology (Fig. 3A,B) and the activation of *th*-Luc (Fig. 3C,D) and other neuronal genes (Fig. 4A). The outgrowth of PC12 neurites was analyzed by measuring the length of neuritic processes using an established assay in cells co-transfected with plasmid expressing marker EGFP protein [23]. Treatment of PC12 cells with NGF produced typical neurite outgrowth (Fig. 3A,B & Fig. S3a, supplementary material). In a loss of function experiment we co-transfected PC12 cells with dominant negative mutants of FGFR1, which lack the tyrosine kinase (TK-) domain, form non-functional dimers with the endogenous receptor and compete with wild type FGFR1 for its nuclear targets [23]. FGFR1(TK-) localizes to cytoplasmic membranes and cell nuclei. FGFR1(SP−/NLS)(TK-), in which the signal peptide (SP-) is replaced with a NLS, functions exclusively in the nucleus [23], [26], [36], [42]. Cells co-transfected with a control vector display short processes, however, when treated with NGF the processes elongate. The dominant negative receptors have no significant effect on neurite length in non-stimulated PC12 cells compared to controls (Fig. 3B). In contrast, cells transfected with FGFR1 (TK-) or FGFR1 (SP−/NLS) (TK-) fail to extend neurites in response to NGF. In a gain of function experiment, PC12 co-transfected with full length constitutiveFGFR1(SP/−NLS) [26], [27], [34], [42], which contains a functional tyrosine kinase domain, display a marked 3-foldelongation of neurites indistinguishable from that induced by NGF (Fig. 3A, B). The effects of FGFR1 on neurite outgrowth are summarized in Fig. 3B.

Additional experiments show that NGF increases the number of PC12 cells with elongated process while reducing the cell number with short (i.e. less than 1 cell body diameter in length) processes. This effect is diminished by dominant negative FGFR1 (Fig. S3B supplementary material). Lastly, transfection of constitutive nuclear active FGFR1(SP−/NLS) also promotes neurite regeneration in the absence of additional NGF stimulation (Fig. S3C supplementary material), indicating the ability of FGFR1 to trigger the mobilization of “primed” intermediaries necessary for neurite formation [44]. Considering all of these results together, we conclude that nuclear FGFR1 signaling is required and sufficient for NGF-induced de novo and regenerative neuritogenesis.

Another frequently used event marking NGF-induced PC12 differentiation is the up-regulation of TH, the rate-limiting enzyme in catecholamine synthesis. The effect exerted at the level of gene transcription is mediated through the proximal *th* gene promoter as a result of binding diverse ssTF that interact with co-activator CBP and its partner FGFR1 [60]. To determine whether NGF activates the *th* gene promoter via nuclear FGFR1, we transfected PC12 cells with a *th* promoter-luciferase reporter plasmid along with dominant negative FGFR1(TK-) or control vectors. Fig. 3C illustrates dose-dependent stimulation of *th* gene promoter activity by 3, 10 and 30 ng/ml NGF. The *th*-Luc expression in the presence of NGF was significantly reduced by FGFR1(TK-) (2.5- to 3-fold reduction) and FGFR1(SP−/NLS)(TK-) (1.4- to 2-fold reduction). The smaller effect of FGFR1(SP−/NLS)(TK-) may reflect its lower expression level. Expression levels of FGFR1(SP−/NLS) or FGFR1(SP−/NLS)(TK-) are typically similar to endogenous FGFR1 while FGFR1(TK-) is expressed at a 2–3 fold higher level (not shown).Dominant negative FGFR1(SP−/NLS)(TK-) had no effect on *th*-Luc expression in the absence of NGF stimulation. Changes in low basal *th*-Luc expression observed in FGFR1(TK-) transfected cells did not attain statistical significance. In contrast, co-transfection of FGFR1(SP−/NLS) produced a statistically significant 2-fold (p<0.001) increase in *th*-Luc promoter activity (Fig. 3D). FGFR1(SP−/NLS)- and NGF-induced increases were not additive suggesting a common mechanism of stimulation. In conclusion, NGF-induced nuclear accumulation of FGFR1 is both necessary and sufficient to up-regulate *th* gene promoter activity.

### Global RNA Synthesis in PC12 Cells is Influenced by Endogenous FGFR1

RNA-SAGE analyses showed that the NGF induced PC12 differentiation is accompanied by an up-regulation of a several genes (>800) and down-regulation of smaller number of genes [61]. Our RT-qPCR analyses confirmed the up-regulation of neuronal *th, dcx* and *βIII-tubulin* genes and additionally showed NGF-induced upregulation of neurogenic *fgf-2, fgfr1,nurr1* and *nur77* (Fig. 4A).

The increase in *dcx* mRNA (Fig. 4A) was accompanied by a 12-fold increase in DCX protein content in individual cells measured by immunocytochemistry and quantitative confocal microscopy (Fig. 4B). DCX up-regulation by NGF was reduced (>70%) by constitutive nuclear FGFR1(SP−/NLS)(TK-). In contrast, basal DCX expression was not affected by FGFR1(TK-). In a gain of function experiment we transfected PC12 cells with full length functional FGFR1(SP−/NLS). FGFR1(SP−/NLS) induced a 3.8-fold DCX-IR increase in the absence of NGF stimulation, however, this effect was not additive with the NGF-induced increase. Thus, while NGF up-regulation of DCX requires nuclear FGFR1, factors in addition to nuclear FGFR1 are required to attain maximal stimulation. Consistent with RNA-SAGE results, which demonstrate widespread activation by NGF [61], we observed an NGF-induced increase in new RNA synthesis using a 5′-Fluoro-Uridine (FU) incorporation assay(Fig. 4C). The short-term (25 min) nuclear incorporation of this halogenated nucleotide analog into nascent newly synthesized RNA was visualized by immunocytochemistry. The intensity of FU immunofluorescence in the individual cell nuclei of transfected (EGFP-positive) cells was measured using quantitative confocal microscopy (Fig. 4C). NGF treatment increased the overall nuclear content of FU-RNA in PC12cells by approximately 45%. In cells transfected with dominant negative FGFR1(TK-) and FGFR1(SP−/NLS)(TK-) the FU labeling of newly synthesized RNA was significantly diminished in non-treated (−30% and –40%, respectively) as well as in NGF-treated cells (−35% and −25%, respectively), indicating that nuclear FGFR1 supports global gene transcription in PC12 cells in the absence and presence of NGF stimulation. Thus, by measures of general transcription activity (5′FU incorporation) FGFR1 plays a prominent role in global RNA synthesis.

### FGFR1 and Nur Binding to NGF Activated Genes

Our recent studies demonstrated that nuclear FGFR1 forms complexes with Nurr1 and Nur77 proteins in mESC and in the rat brain [24], [25]. The expression of Nur proteins in PC12 cells was verified using anti-Nur77/Nurr1 (Santa Cruz), which recognizes either of these related proteins. Nur77/Nurr1are represented by 65 and 58 kDa bands (Fig. S4, supplementary material). Treatment of PC12 cells with NGF results in the transient up-regulation of a 65 kDa cytoplasmic protein by the 2^nd^ day of treatment. NGF treatment appears to have little effect on the total steady-state levels of nuclear Nur77/Nurr1.

**Figure 5:**
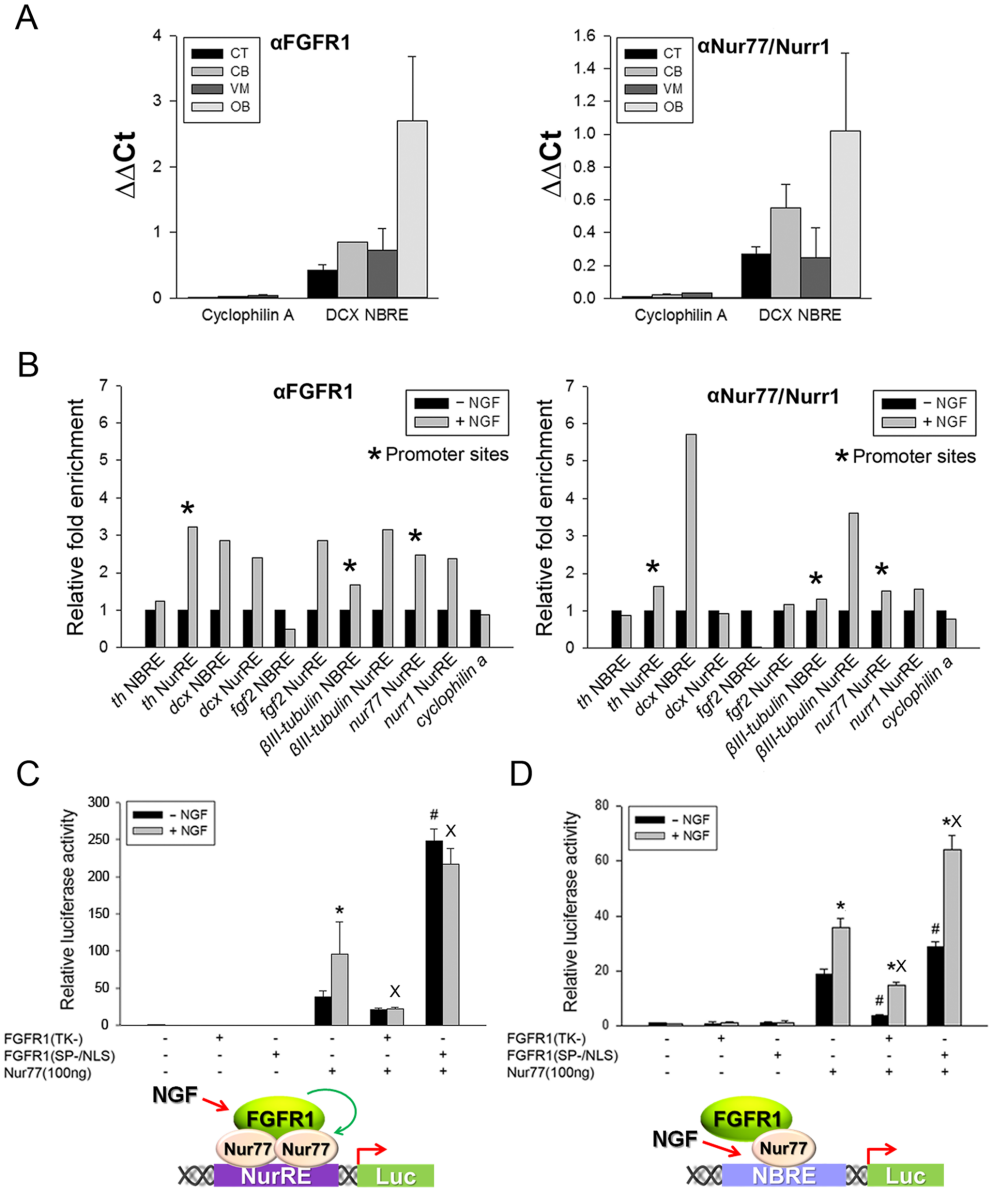
NGF stimulates FGFR1 binding to NGF-activated genes and cooperates with FGFR1 in activation of Nur-responsive elements - NuRE and NBRE. (**A**) In vivo validation of FGFR1 and Nur binding to an NBRE-like site within intron 1 of the DCX gene. Chromatin was immunoprecipitated with FGFR1, Nur77/Nurr1Ab or IgG in dissected rat brain regions including, the olfactory bulb (OB) which express the *dcx* gene at high levels [62], and in the cortex (CX), cerebellum (CB) and ventral midbrain (VM), which express the *dcx* gene at lower levels. The *cyclophilin A* gene, which lacks potential Nur-binding NurRE, NBRE or RARE like elements was used as a control. Samples were combined from two rats and the graphs show ΔΔCt means±SEM of triplicate assays. (**B**) PC 12 cells were incubated with or without NGF for 48 hours. ChIP-qPCR was performed with FGFR1 or Nur77/Nurr1 antibodies or control IgG within diverse NGF-activated genes. Similar as in vivo, no binding to the cyclophilinA gene was observed (not shown). Graphs show means of duplicate or triplicate samples from a representative experiment. Similar changes were observed in three separate experiments. (**C,D**) FGFR1 augments Nur77 NurRE- and NBRE-dependent transcription in PC12 cells. Cells were transfected with NurRE-Luc (**C**) or NBRE-Luc (**D**) and either FGFR1(SP−/NLS) or dominant negative FGFR1(TK-) in the presence or absence of Nur77. The amount of transfected DNA was adjusted to 1 µg per well. One day after transfection, PC12 cells were incubated with NGF (50 ng/ml) or without NGF for an additional 24 hours. Results represent the mean ± SEM from 2 experiments with at least three replicates. One-Way ANOVA, LSD: *p<.001 difference between –NGF and +NGF in individual plasmid transfection groups; #p<.001 difference between Nu77 and Nur77+ FGFR1(SP−/NLS) (or FGFR1(TK-) in the absence of NGF; ^X^p<.001 difference between Nu77 and Nur77+ FGFR1(SP−/NLS) (or FGFR1(TK-) in the presence of NGF. Inserts show the proposed mechanisms of FGFR1 and Nur77 co-operation during NGF activation, including dimer binding NuRE and monomer-binding NBRE.

In each of the NGF activated genes (Table 1) there are regions containing the AGGTCA sequence. This is similar to both the NBRE motif, targeted by monomeric Nur77 and Nurr1, and to the NurRE, which binds Nur homo or heterodimers. We previously showed FGFR1 and Nur binding to the *th* gene promoter and intron regulatory regions in the rat brain substantia nigra [25]. Similar binding occurs in mESC and is regulated by RA [24]. Here, we first verified specific DNA-sequence dependent FGFR1 binding to the *dcx* gene in vivo and in PC12 cells after NGF induction. In the *dcx* gene, intron 1 contains two elements with sequences homologous to the NBRE and NurRE consensus sites. FGFR1 and Nurr1 binding to this NBRE sequence was investigated in DCX-expressing olfactory bulb (OB) and brain regions which show little or no DCX expressing neuroblasts: ventral midbrain (VM) region, cerebellum, and cortex [62]. The strongest binding occurred in the OB, consistent with highest expression of DCX in this brain structure [62]. A similar pattern of binding was observed with other Nur proteins. This tissue pattern of FGFR1 and Nur binding to the *dcx* gene differs from the FGFR1 and Nur binding to the *th* gene, which was most pronounced in the ventral midbrain region which contains *th*-expressing dopamine neurons [25]. For control DNA, we used an intragenic region of the *cyclophilinA* gene which includes fragments of exons 2 and 3 and intron 2, but lacks putative NBRE or NurRE sites. No specific FGFR1 or Nur binding was detected at the *cyclophilinA* gene region in either of the examined brain tissues (Fig. 5A).

We next analyzed the in vitro binding of FGFR1 and Nur77/Nurr1 to potential Nur target sites in all genes that were up-regulated by NGF in PC12 cells. In addition to the *dcx* and *th* genes, we identified potential Nur-interacting sequence motifs in the *βIII-tubulin, nur77, nurr1,* and *fgf2* genes (Fig. 5B; Table 1).NGF increased FGFR1 and Nur binding to the NurRE-like site in the *th* gene promoter but did not affect binding to the NBRE in intron 1. In intron 1 of the *dcx* gene, NGF increased FGFR1 binding to both the NBRE and NurRE sites, but only increased Nur binding to the NBRE. In the *βIII-tubulin* gene, NGF stimulation increased FGFR1 binding to the promoter region containing an NBRE-like site and to intron-1 containing a NurRE-like element. NGF increased Nur77/Nurr1 binding only at the intron NurRE region. NGF also increased FGFR1 binding to NurRE containing regions in the *nur77* gene promoter and in intron-1 of the *nurr1* gene. Binding of Nur77/Nurr1 to those regions showed only minimal effect of NGF. In the fgf-2 gene, both NurRE- and NBRE-like sites are located in intron-1. NGF stimulation increased FGFR1 binding to the NurRE element and reduced binding to the NBRE. The binding of Nurr1/77 to the NurRE was reduced while binding to the NBRE was unaffected by NGF. Thus, NGF increases FGFR1 binding to Nur-like target motifs in all of the NGF-stimulated genes. Interestingly, the binding pattern of Nurs mirrors the binding pattern of FGFR1 or is unchanged by NGF stimulation.

### Nuclear FGFR1 and Nurs Co-activate Transcription of NurRE and NBRE Elements in PC12 Cells

To determine whether FGFR1 and Nurs can act cooperatively at isolated target motifs we examined transactivation of luciferase-linked NurRE (Fig. 5C) and NBRE (Fig. 5D), which bind Nur dimers and monomers, respectively. We conducted experiments in which effects of co-transfected pCDNA3.1 plasmids expressing different proteins from the CMV promoter were examined in the absence or presence of NGF treatment. Ina pilot experiment we determined that NGF did not affect the expression of control EGFP protein from the CMV promoter (not shown).

Fig. 5C shows approximately 40-fold activation of the NurRE-Luc by Nur77.In the absence of co-transfected Nur77, FGFR1(SP−/NLS) had no effect on the NuRE promoter activity. However,co-transfectedFGFR1(SP−/NLS) potentiated Nur77stimulation nearly 7-times to 260-fold. In contrast, dominant negative FGFR1(TK-) reduced Nur77 mediated activation by 50%.LikeFGFR1(SP−/NLS), NGF also stimulated NurRE-Luc ranscription and this stimulation was dependent on the co-expression of Nur77. The Nur77-dependent NGF stimulation was blocked by dominant negative FGFR1(TK-) and was not additive with the stimulation by constitutive active nuclear FGFR1(SP−/NLS) (Fig. 5C). In the same experiments, Nur77 also markedly enhanced (∼20-fold) NBRE-dependent luciferase transcription (Fig. 5D). However, the additional stimulation of Nur77-NBRE-Luc by NGF (1.9-fold) or FGFR1(SP−/NLS) (1.5-fold) was less pronounced than the stimulation of the Nur77-NuRE-Luc (Fig. 5C).In the presence of NGF, FGFR1(SP−/NLS) maintained its stimulating effect by bringing the overall increase in Nur77-NBRE-Luc activity to 3.4-fold. ANOVA (2-way) revealed a statistically significant interaction between NGF and FGFR1(SP−/NLS) in Nur77-NBREstimulation (p = 0.019), indicating separate yet interactive activation mechanisms. Consistent with this outcome, FGFR1(TK-) diminished Nur77-dependent NBRE activity both in the absence (−80%) and presence of NGF (−60%), but did not abolish NGF stimulation. Thus, while endogenous FGFR1 is required for NGF activation of Nur77 dimers on the NurRE (Fig. 5C), the nuclear FGFR1 function on the NBRE involves an up-regulation of basal Nur77 monomer activity (Fig. 5D).

Nurr1 activation of NurRE-dependent transcription was also augmented by NGF and nuclear FGFR1(SP−/NLS) in PC12 cells (Fig, S5A), albeit to a markedly lesser extent than NurRE activation by Nur77 (compare to Fig. 5C). NGF increased Nurr1 stimulation of NBRE-Luc3-fold but was not significantly augmented by FGFR1(SP−/NLS) (Fig. S5B). In conclusion,FGFR1 mediates NGF activation of Nur77 dimers less effectively that of Nurr1 dimers. Nuclear FGFR1 activates the basal activity of monomeric Nur77 (but not Nurr1) through a mechanism that appears to be independent of NGF activation.

## Discussion

NGF has been shown to set in motion and provide chronic regulation of diverse actions that include many features of the neuronal phenotype, such as gene reprogramming and neuritogenesis. This process is controlled by activating RSK and Nur77, which are also central features of the INFS mechanism. Our results demonstrate that INFS contributes unique and previously unknown requirements to support specific end-points of the NGF mechanism, including gene activation and neurite outgrowth. These results help to explain how NGF signal transduction activates Nur-dependent gene activities which underlie neuronal differentiation.

**Figure 6:**
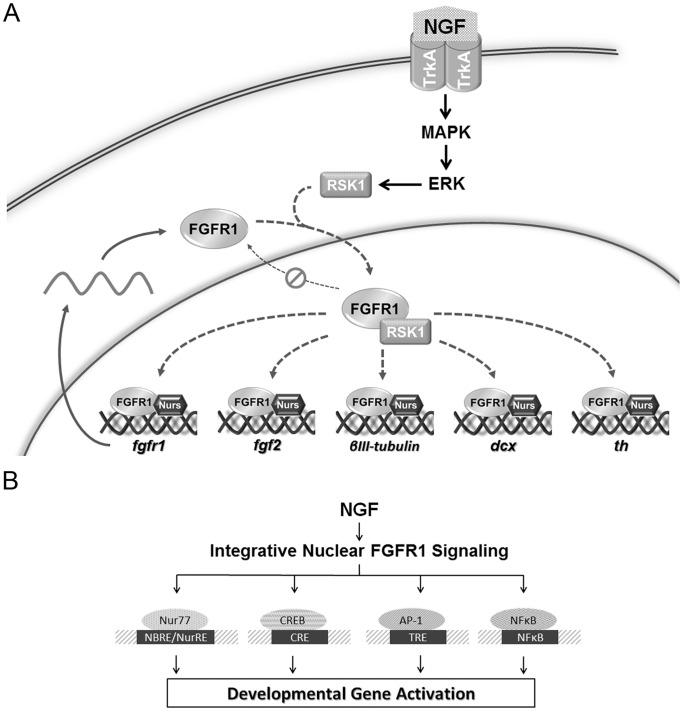
INFS as a transducer of NGF signals to ssTF. (**A**). Cross-talk between NGF signaling and INFS. NGF stimulates the MAP/ERK pathway resulting in increased RSK1 activity [11], [13], which is known to promote the release of newly synthesized FGFR1 from pre-Golgi membranes into the cytosol and accumulation in the cell nucleus [33]. RSK1 binding to FGFR1 in the nucleus decreases FGFR1 mobility promoting the FGFR1 nuclear retention accumulation [32]. Increased nuclear FGFR1 correlates with FGFR1 interaction with Nur proteins [24], [25] and Nurs also decrease nuclear FGFR1 movement [25]. The FGFR1/Nur complexes generated by NGF stimulation activate Nur-responsive sites on diverse genes. In addition, upregulation of *fgf-2* mRNA by NGF (Fig. 4A) could also contribute to FGFR1 nuclear trafficking since the nuclear accumulation and retention of FGFR1 is facilitated by its ligand FGF-2 [32], [36] (not shown on diagram). (**B**) The end-point ssTF of NGF stimulation, in addition to Nurs, include CREB, AP1, and NFKB [71]. The same end-point factors were shown to be activated by nuclear FGFR1 through the CBP/RSK1-dependent feed forward mechanism [23]. This common INFS step may enable coordinated programing of diverse genes necessary for NGF-induced neuronal differentiation.

A central and essential functional feature of INFS is the nuclear accumulation of FGFR1. The present study verifies that the nuclear accumulation of FGFR1 constitutes a common response to NGF in both neural crest derived rat PC12 and human neuroblastoma cells. In live cells FLIP studies demonstrate nuclear and cytoplasmic FGFR1 are in kinetically distinct, yet connected cellular compartments. NGF promotes FGFR1 nuclear accumulation by reducing FGFR1 nuclear export, adding to other established mechanisms of FGFR1 nuclear accumulation:(i) generation of cytosolic, rapidly diffusing FGFR1 facilitated by FGFR1 binding proteins, RSK1 and NLS-containing 23 kDa FGF-2 [32], [33], (ii) importin-β-mediated nuclear transfer [34] and (iii) regulation of intranuclear FGFR1 mobility [24], [32], [33]. Thus, there are several potential regulatory mechanisms through which NGF influences both the nuclear import and export of FGFR1 and FGFR1-dependent gene regulation. Fig. 6A summarizes the juxtaposition of NGF signaling and INFS activation. NGF stimulates the MAP/ERK pathway resulting in increased RSK1 activity [11], [13], which is known to promote FGFR1 release from cytoplasmic pre-Golgi membranes and generate high mobility cytosolic receptor that accumulates in the cell nucleus [33]. Nuclear accumulation of FGFR1 may be facilitated by its NLS-containing ligand FGF-2 [32], suggested by NGF upregulation of *fgf-2* mRNA (Fig. 4A). RSK1 binding to FGFR1 in the nucleus decreases FGFR1 mobility, further promoting the nuclear accumulation of FGFR1 [32]. Nuclear accumulation of FGFR1 correlates with FGFR1-Nur binding [24], [25], in which Nurs restrict the intranuclear movement of FGFR1 [25]. Thus, the FGFR1 interaction with RSK1 and Nurs may underlie the NGF inhibition of FGFR1 nuclear export, shown by our FLIP experiments, and the NGF-induced nuclear accumulation of FGFR1. Finally, the FGFR1-Nur cooperation at the Nur-targeted DNA sites transduces gene activation by NGF. Together these observations offer mechanistic support for INFS mediated action of NGF.

The causal relationship between NGF-induced PC12 cell differentiation and nuclear accumulation of FGFR1 was demonstrated by measuring neurite outgrowth, the principal morphological consequence of NGF stimulation. The importance of endogenous nuclear FGFR1 for neuronal differentiation is underscored by the observation that dominant negative tyrosine kinase deleted nuclear FGFR1(SP−/NLS)(TK-) or nuclear/cytoplasmic FGFR1(TK-) [1], [23], [26], [36] prevents NGF-induced neurite outgrowth and transcriptional programming (i.e., diminished DCX expression, *th* promoter activity and overall RNA synthesis in NGF stimulated cells). Conversely, transfection of constitutively active nuclear FGFR1(SP−/NLS) initiates neurite outgrowth, neurite regeneration and activates transcription in the absence NGF. This demonstrates nuclear accumulation of FGFR1 constitutes a sufficient signal for activation of neuronal genes as well as initiation and maintenance of morphological differentiation. The extent of this differentiation will be investigated further to delineate the associated physiological features. Activation of INFS by transfection of nuclear FGFR1 was shown to induce an exit from the cell cycle, morphological differentiation and the expression of neuron-specific proteins in PC12 cells (present study), human brain- [1] or umbilical cord blood-derived Neural Progenitor Cells [23], mESC [24], neoplastic medulloblastoma and neuroblastoma cells [1], [23], [29]. Furthermore, transfection of nuclear FGFR1 or its 23 kDa FGF-2 ligand re-instates neuronogenesis in the adult brain in vivo [29], [63] (reviewed in [28], [30], [64]). Our results are consistent with earlier demonstrations of NGF up-regulation of FGFR1 expression and FGFR1 activation in PC12 cells [65], [66], as well as the inhibition of NGF-induced differentiation by dominant negative FGF receptors.

Gene expression is clearly re-ordered during NGF-induced neuronal differentiation [61] and the present investigation demonstrates the involvement of INFS in this aspect of NGF signaling. The gene responsible for the rate-limiting enzyme for catecholamine synthesis, *th*, is activated by nuclear FGFR1 in cooperation with CBP [23], [36]. Transfection of constitutive nuclear FGFR1(SP−/NLS) augmented *th* promoter activation by NGF in luciferase reporter assays. The role of endogenous FGFR1 in *th* promoter regulation is revealed by the dose-dependent inhibition of NGF activation of the *th*-Luc promoter in the presence of dominant-negative FGFR1(SP−/NLS)(TK-) and FGFR1(TK-). The inhibitory function of dominant negative FGFR1 relies mainly on competitive binding of the mutant to the CBP/RSK complex, preventing the release of CBP from this inactive complex [23].

The transcription of *th* as well as other NGF stimulated genes, *dcx*, *fgfr1*, *fgf2*, *βIII-tubulin*, *nurr1* and *nur77*, appears to be stimulated by NGF and involves gene binding by nuclear FGFR1 and Nurs. NGF treatment up-regulates *nur77* and nurr1 *mRNA*, increases the protein content of Nur proteins and increases FGFR1 as well as Nur binding to several NGF stimulated genes in PC12 cells. These findings are consistent with the proposed functions of Nur77 as an intermediary in NGF-induced gene expression [12], and suggest a similar role for Nurr1.

NGF increased Nur binding to an NBRE-like region in the *th* and *βIII-tubulin* gene promoters and in intron-1 of the *dcx* gene. In addition, NGF increased Nur binding to NurRE-like sites within the *nur77* promoter and within introns of *βIII-tubulin* and *nurr1*. At these loci, while Nur binding was not increased by NGF (*dcx*-NurRE, *fgf2*-NurRE), we observed an NGF-induced increase of FGFR1 binding, suggesting that nuclear FGFR1 provides stimulation of Nur-dependent transcription. This model is supported by our experiments with isolated NurRE and NBRE elements in which overexpressed nuclear FGFR1 augmented Nur77-dependent transcription. The verified binding of FGFR1 and Nur to the *dcx* (present study) and *th* gene [25] in brain tissues indicates that this mechanism also operates in vivo in a brain region-specific manner.

The present luciferase reporter assays, employing isolated NurRE and NBRE reporter elements, demonstrate a cooperative function of Nur77 and nuclear FGFR1 in transcriptional activation of Nur-binding DNA sites in PC12 cells. In the presence of Nur77, nuclear FGFR1 is sufficient to activate NurRE to a lesser degree than NBRE. Furthermore, dominant negative FGFR1(TK-) inhibits the activation of NBRE and NurRE induced by Nur77, indicating the participation of endogenous FGFR1 in Nur77-dependent transcription. These experiments indicate that nuclear FGFR1 works together with Nur77 during NGF gene stimulation. At the NurRE site, occupied by overexpressed Nur77 dimers, nuclear FGFR1 supports basal Nur77 activity and NGF stimulation. In contrast, on the NBRE, which binds Nur77 monomers, the role of nuclear FGFR1 appears limited to supporting basal Nur77 activity without being necessary for NGF stimulation (Fig. 5C and D - models). Thus, NGF and FGFR1 act in complementary fashion in activating the Nur77 monomer. Up-regulation of DCX and NGF induced neurite outgrowth were blocked by dominant negative FGFR1 and is consistent with Nur77 dimer-dependent regulation.

Importantly, INFS activation provides a mechanism sufficient to promote Nur77 dependent gene activation in the absence of NGF, similar to what has been observed for PC12 cell morphological differentiation. Therefore, we propose that FGFR1 serves as a co-factor for Nur-mediated gene activation and differentiation. In general, nuclear FGFR1 activation of Nur77 is more pronounced than Nurr1 in PC12 cells, consistent with the model in which Nur77 transduces NGF stimulation [12].

A principal finding of our investigation is the cooperative function of Nurs and nuclear FGFR1 on genes related to PC12 neuronal differentiation. Both FGFR1 and Nur77/Nurr1 are central nuclear integrators of diverse developmental signals and have been implicated in post-mitotic development. Our previous work has identified the association of FGFR1 with retinoid and orphan Nur nuclear receptors on regulatory regions on *th*, *fgf2* and *fgfr1* genes in mESC [24], and on the *th* gene in the rat brain [25]. The mechanisms by which nuclear FGFR1 increases Nur transcriptional activity also require further investigation. Through interactions with CBP, FGFR1 may recruit this transcriptional co-activator to Nur-occupied DNA enhancers. Such a mechanism appears to operate during RA-induced gene activation in which coordinated binding of Nur, FGFR1 and CBP to RA-activated genes is observed [24]. Another possible mechanism could involve post-translational modifications of Nurs that may be promoted by FGFR1. Nurs can be regulated by phosphorylation as well by acetylation [67], [68], [69], [70]. For instance, Nurr1 phosphorylation by ERK2 plays an important role in regulating TH expression [70]. A recent report that the acetylation by CBP-related p300, and the HDAC1 partner, increase the stability of Nur77, further suggesting a mechanism by which various factors, including NGF, may control Nur77 turnover and function [68]. Thus, activation of INFS, which leads to the dissociation of an inactive CBP-RSK1 complex and the subsequent activation of CBP and RSK1 by FGFR1 [22], [23], may provide a stimulating effect for Nur-dependent transcriptional activation.

Our results provide upstream context for the previously demonstrated essential role of RSK1 in NGF-induced PC12 neuronal differentiation [13]. The tyrosine kinase domain of nuclear FGFR1 directly binds and activates RSK1 [22], and may link the NGF receptor stimulation to RSK1. Consistent with this proposed link, both dominant negative nuclear FGFR1 (present study) and RSK1 knock down [13] prevent NGF-mediated PC12 neurite outgrowth.

NGF acts via multiple ssTF including CREB, AP1 and NfkB [71], which also are activated by nuclear FGFR1 as shown in our earlier studies [23]. Therefore, we propose that INFS acts as universal transducer of NGF initiated signals to diverse ssTF (Fig. 6B). This common INFS step may enable coordinated programing of diverse genes necessary for NGF-induced neuronal differentiation. Potential perturbation of the INFS mechanism, brought about by gene binding of truncated FGFR1 [43], could underlie pathological gene reprogramming in cancer cells.

Finally, our findings shed new light on the function of “fibroblast growth factors” in development. Proteins that carry historical names of FGFs and FGF receptors are not found in single celled organisms but are common to diverse multicellular animals suggesting the generation of specialized cells require FGFs. Indeed, knock out (KO) of ubiquitous *fgfr1* prevents gastrulation, while conditional KO in specific tissues blocks development [30]. Of particular note in the evolution of the FGF family is an inclusion of a NLS or the acquisition of a cleavable secretion SP. While secreted FGFs (i.e. 18 kDa FGF-2) act as mitogens, NLS-containing High Molecular Weight (23 kDa) FGF-2 is a differentiation-promoting factor that acts in the cell nucleus [27], [28]. Similarly, in the evolution of FGF receptors (R1–4) one observes adaptations that direct these proteins to different cellular compartments. Typically, a α-helical, hydrophobic TMD anchors FGFR4 in the lipid bilayer generating a membrane-integrated receptor which interacts with secreted FGFs [31]. In contrast, a newly synthesized FGFR1 is either incorporated into cytoplasmic membranes or released from the endoplasmic reticulum, enabled by an atypical β-sheet, hydrophilic TMD [31], generating a high mobility cytosolic protein [33] which enters the cell nucleus [28]. Thus, FGFs and FGFR appear to have evolved as intracellular (nuclear) or extracellular (released FGFs and plasma membrane FGFR) signaling systems. Nuclear functions of FGFs appear early in evolution, as indicated by the presence of NLS residues in the FGF-related LET-756 in C. elegans, and by the targeting of LET-756 to presumed transcription sites [72].

In summary, our investigation expands the notable integrative functions of INFS to include NGF, the first discovered pluripotent neurotrophic factor. The neuronal differentiation promoting function of gene binding nuclear FGFR1 is fundamentally important for understanding normal development. This novel FGFR1 function can be used for devising and implementing regenerative therapeutic interventions for diverse neurological diseases.

## Supporting Information

Figure S1 **Nuclear accumulation of FGFR1 is revealed by different FGFR1 antibodies and is facilitated by Leptomycin B (LMB). (A)** PC12 cells were treated with 50 ng/ml NGF for the indicated time periods or maintained in 1% horse serum control culture medium. Immunostaining with C-terminal polyclonal rabbit αFGFR1 (Santa Cruz) plus goat anti-rabbit Alexa 568 confirms NGF induces morphological changes and the nuclear accumulation of FGFR1. **(B)** PC12 cells were treated with 50 ng/ml NGF for the indicated time period or maintained in 1% horse serum control culture medium. Immunostaining with mouse monoclonal αFGFR1 (McAb6) plus goat anti-mouse Alexa 488 confirms NGF induces morphological changes and the nuclear accumulation of FGFR1. **(C)** LMB facilitates the nuclear accumulation of FGFR1. PC12 cells were treated with 50 ng/ml NGF and 100 ng/ml LMB (0.1% v/v ethanol) for 2 hr or maintained in control culture medium. Immunostaining with mouse monoclonal αFGFR1 (McAb6) plus goat anti-mouse Alexa 488 confirms LMB facilitates the nuclear accumulation of FGFR1 by blocking the nuclear export of FGFR1.
(TIF)

Figure S2 **NGF increases intracytoplasmic FGFR1 mobility and reduces FGFR1 nuclear>cytoplasmic export – FLIP analysis. (A)** FGFR1-EGFP was transfected into PC12 cells. Twenty-four hours after transfection, cultures were maintained in medium containing 1% horse serum or were additionally treated with 50 ng/ml NGF for 48 h followed by confocal imaging. An example of a FGFR1-EGFP expressing cell before and after photo-bleaching is shown. A region of the cytoplasm outside Golgi vesicles (red rectangle) was bleached by high intensity laser as described in Materials and Methods and the loss fluorescence intensity was analyzed in the nucleus (blue rectangle) and in the cytoplasm (yellow rectangle). **(B)** Single-exponential analysis of FGFR1-EGFP FLIP regression in representative cells. **(C)** Single-exponential analysis of FGFR1-EGFP FLIP regression in the cytoplasm (n>7) shows that NGF inhibits FGFR1-EGFP trafficking between the nucleus and cytoplasm. The rate of cytoplasmic FGFR1-EGFP fluorescence loss after another region of the cytoplasm was bleached corresponds to half time = 86.84, and in NGF treated cells to half time = 57.43 sec. However, this 1.5-fold difference did not approach statistical significance (p>0.1). The rate of the nuclear ROI fluorescence loss was 270.92 sec, at least 3-times slower than in the cytoplasm (p<0.05). In contrast, the rate of nuclear FGFR1-EGFP loss after cytoplasmic photobleaching (half time = 270.92 sec) was markedly reduced by NGF (in all NGF treated cells the half time was greater than 10,000 sec and can no longer be effectively measured during the entire duration of the experiment.
(TIF)

Figure S3 **Neurite outgrowth and regeneration in PC12 cells are mediated by nuclear FGFR1. (A)** The time dependent elongation of neurite outgrowth induced by NGF in PC12 cells. PC12 cells were treated with 50 ng/ml NGF for an indicated time period or maintained in 1% horse serum control culture medium. After cells were imaged by using light microscope, the longest process in an individual cell was measured. (**B)** Dominant negative FGFR1 abolishes neurite outgrowth. PC12 cells were transfected with two plasmids, one expressing the dominant negative FGFR1 mutant or control pcDNA3.1 and the second expressing EGFP. EGFP diffuses throughout the cell permitting visualization of the entire neuritic network. Twenty-four hours after transfection cultures were treated with 50 ng/ml NGF for an additional 10 days and imaged using fluorescent microscopy. Both dominant negative FGFR1 mutants, FGFR1(TK-) and FGFR1(SP−/NLS)(TK-), significantly reduce the percentage of neurite outgrowth induced by NGF. At least 30 cells were calculated in each transfection group. **(C)** Regeneration of PC12 cells neurites**.** Three separate equivalent groups of PC12 cells were cultured in the presence of NGF for 20 days, followed by transfection with either EGFP alone, EGFP+FGFR1(SP−/NLS) or EGFP+FGFR1(TK-). Two days after transfection, NGF was removed from all of the cultures by trituration and repeated washing and re-suspension in RPMI medium +1% horse serum (no NGF) and centrifugation. The cells from each group were next divided in half and re-plated on collagen-coated culture dishes either with or without NGF. Those cells that were successfully transfected (viewed under fluorescence imaging for EGFP expression) were scored for neurite extension (greater than 2 cell body diameters) 21 hours later. The regeneration results (% of total green cells with measureable neurites) are illustrated by the bar graph.
(TIF)

Figure S4 **NGF-induced upregulation of Nur77/Nurr1 in PC12 cells.** Western blotting revealed transient NGF-induced increases in Nur77/Nurr1 protein bands in the cytoplasm and in the nucleus. Pan Nur77/Nurr1 antibody (Santa Cruz) was used and the signals were normalized to GADPH (cytoplasmic) and matrin-3 (nuclear).
(TIF)

Figure S5 **Nuclear FGFR1 and NGF augment Nurr1-mediated transcription.** **(A)** PC12 cells were transfected with a NurRE-Luc reporter and Nurr1, FGFR1(SP−/NLS) or control β-galactosidase and treated with NGF or control medium for 6 h. FGFR1(SP−/NLS) has no significant effect on NurRE-Luc activation in the absence of Nurr1. However, the transcriptional activity of Nurr1 on the NurRE reporter is strongly enhanced by nuclear FGFR1(SP−/NLS) in both NGF treated and non-treated conditions (One-Way ANOVA *p<.001 different to no NGF treatment in individual groups; #p<.001 different to Nurr1 only; ^X^p<.001 different to Nurr1+ NGF). **(B)** PC-12 cells were transfected with a NBRE-Luc reporter and with Nurr1, FGFR1(SP−/NLS) or control β-galactosidase and treated with NGF or control medium for 24 h. No significant change between control and FGFR1(SP−/NLS) groups in the absent of Nurr1. The transcriptional activity of Nurr1 on the NBRE reporter is only slightly enhanced by nuclear FGFR1(SP−/NLS) after NGF treatment (One-Way ANOVA *p<.001 different to no NGF treatment in individual groups; #p<.05 different to Nurr1+ NGF).
(TIF)

Table S1 **Primers used for qPCR.**
(DOCX)

Table S2 **Primers used for Chromatin Immunoprecipitation (ChIP).**
(DOCX)
