# Supplementary figures and images for: NGF-Induced Cell Differentiation and Gene Activation Is Mediated by Integrative Nuclear FGFR1 Signaling (INFS)
Source: PLoS One. 2013 Jul 10;8(7):e68931. doi: 10.1371/journal.pone.0068931 (PMC3707895; doi:10.1371/journal.pone.0068931)

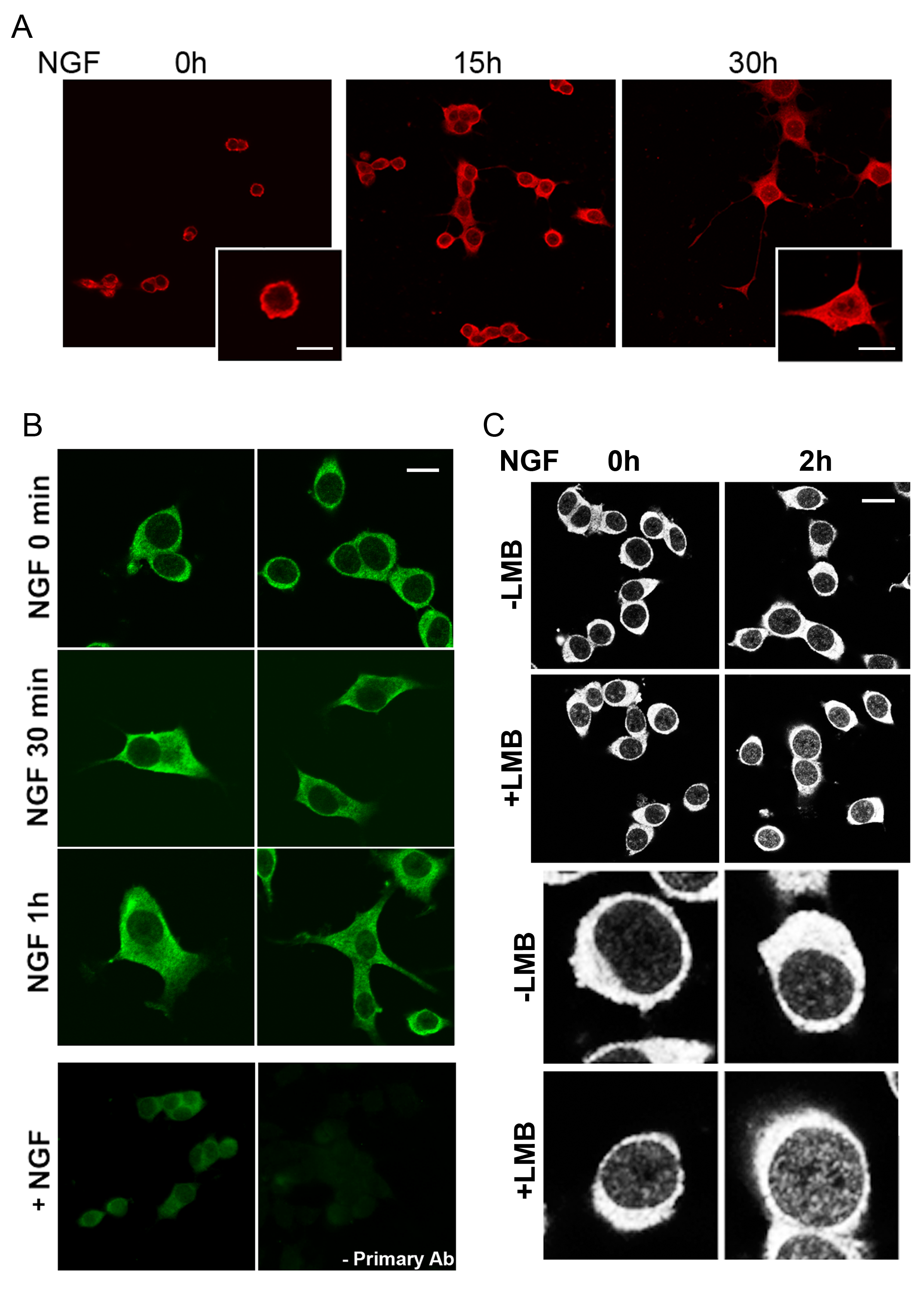

Supplement: Figure S1 — Nuclear accumulation of FGFR1 is revealed by different FGFR1 antibodies and is facilitated by Leptomycin B (LMB). (A) PC12 cells were treated with 50 ng/ml NGF for the indicated time periods or maintained in 1% horse serum control culture medium. Immunostaining with C-terminal polyclonal rabbit αFGFR1 (Santa Cruz) plus goat anti-rabbit Alexa 568 confirms NGF induces morphological changes and the nuclear accumulation of FGFR1. (B) PC12 cells were treated with 50 ng/ml NGF for the indicated time period or maintained in 1% horse serum control culture medium. Immunostaining with mouse monoclonal αFGFR1 (McAb6) plus goat anti-mouse Alexa 488 confirms NGF induces morphological changes and the nuclear accumulation of FGFR1. (C) LMB facilitates the nuclear accumulation of FGFR1. PC12 cells were treated with 50 ng/ml NGF and 100 ng/ml LMB (0.1% v/v ethanol) for 2 hr or maintained in control culture medium. Immunostaining with mouse monoclonal αFGFR1 (McAb6) plus goat anti-mouse Alexa 488 confirms LMB facilitates the nuclear accumulation of FGFR1 by blocking the nuclear export of FGFR1. (TIF) [file pone.0068931.s001.tif]

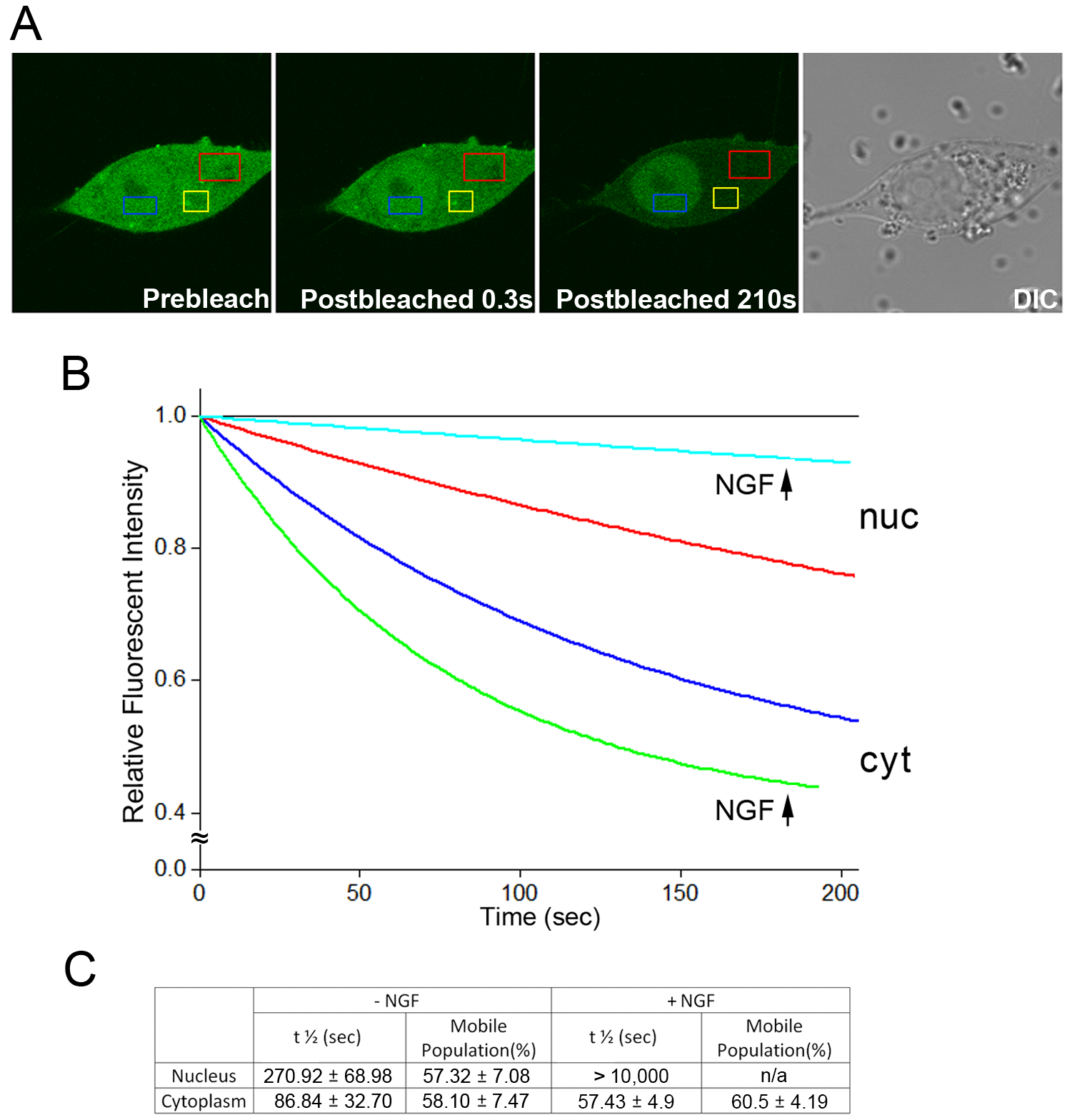

Supplement: Figure S2 — NGF increases intracytoplasmic FGFR1 mobility and reduces FGFR1 nuclear>cytoplasmic export – FLIP analysis. (A) FGFR1-EGFP was transfected into PC12 cells. Twenty-four hours after transfection, cultures were maintained in medium containing 1% horse serum or were additionally treated with 50 ng/ml NGF for 48 h followed by confocal imaging. An example of a FGFR1-EGFP expressing cell before and after photo-bleaching is shown. A region of the cytoplasm outside Golgi vesicles (red rectangle) was bleached by high intensity laser as described in Materials and Methods and the loss fluorescence intensity was analyzed in the nucleus (blue rectangle) and in the cytoplasm (yellow rectangle). (B) Single-exponential analysis of FGFR1-EGFP FLIP regression in representative cells. (C) Single-exponential analysis of FGFR1-EGFP FLIP regression in the cytoplasm (n>7) shows that NGF inhibits FGFR1-EGFP trafficking between the nucleus and cytoplasm. The rate of cytoplasmic FGFR1-EGFP fluorescence loss after another region of the cytoplasm was bleached corresponds to half time = 86.84, and in NGF treated cells to half time = 57.43 sec. However, this 1.5-fold difference did not approach statistical significance (p>0.1). The rate of the nuclear ROI fluorescence loss was 270.92 sec, at least 3-times slower than in the cytoplasm (p<0.05). In contrast, the rate of nuclear FGFR1-EGFP loss after cytoplasmic photobleaching (half time = 270.92 sec) was markedly reduced by NGF (in all NGF treated cells the half time was greater than 10,000 sec and can no longer be effectively measured during the entire duration of the experiment. (TIF) [file pone.0068931.s002.tif]

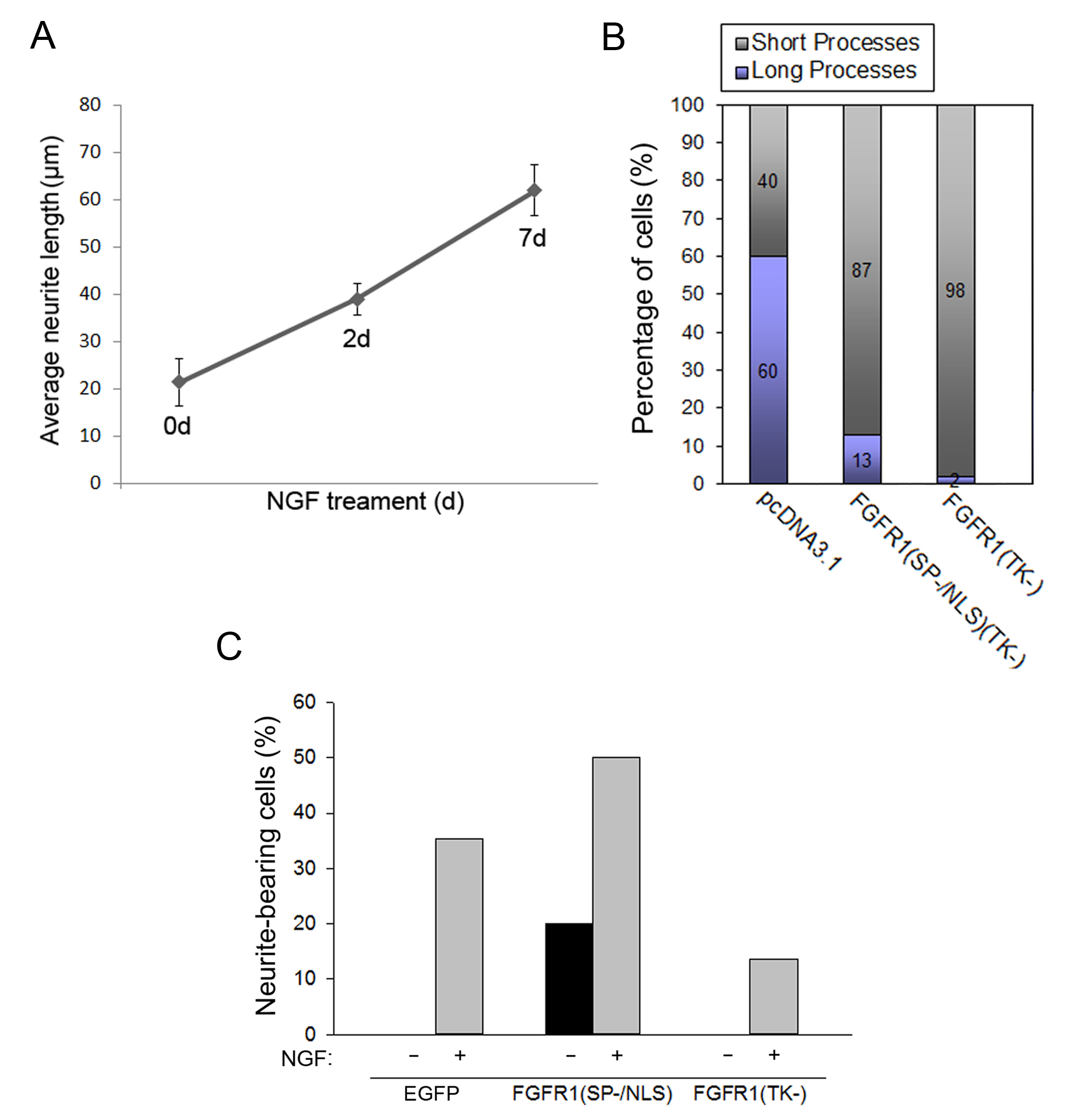

Supplement: Figure S3 — Neurite outgrowth and regeneration in PC12 cells are mediated by nuclear FGFR1. (A) The time dependent elongation of neurite outgrowth induced by NGF in PC12 cells. PC12 cells were treated with 50 ng/ml NGF for an indicated time period or maintained in 1% horse serum control culture medium. After cells were imaged by using light microscope, the longest process in an individual cell was measured. (B) Dominant negative FGFR1 abolishes neurite outgrowth. PC12 cells were transfected with two plasmids, one expressing the dominant negative FGFR1 mutant or control pcDNA3.1 and the second expressing EGFP. EGFP diffuses throughout the cell permitting visualization of the entire neuritic network. Twenty-four hours after transfection cultures were treated with 50 ng/ml NGF for an additional 10 days and imaged using fluorescent microscopy. Both dominant negative FGFR1 mutants, FGFR1(TK-) and FGFR1(SP−/NLS)(TK-), significantly reduce the percentage of neurite outgrowth induced by NGF. At least 30 cells were calculated in each transfection group. (C) Regeneration of PC12 cells neurites. Three separate equivalent groups of PC12 cells were cultured in the presence of NGF for 20 days, followed by transfection with either EGFP alone, EGFP+FGFR1(SP−/NLS) or EGFP+FGFR1(TK-). Two days after transfection, NGF was removed from all of the cultures by trituration and repeated washing and re-suspension in RPMI medium +1% horse serum (no NGF) and centrifugation. The cells from each group were next divided in half and re-plated on collagen-coated culture dishes either with or without NGF. Those cells that were successfully transfected (viewed under fluorescence imaging for EGFP expression) were scored for neurite extension (greater than 2 cell body diameters) 21 hours later. The regeneration results (% of total green cells with measureable neurites) are illustrated by the bar graph. (TIF) [file pone.0068931.s003.tif]

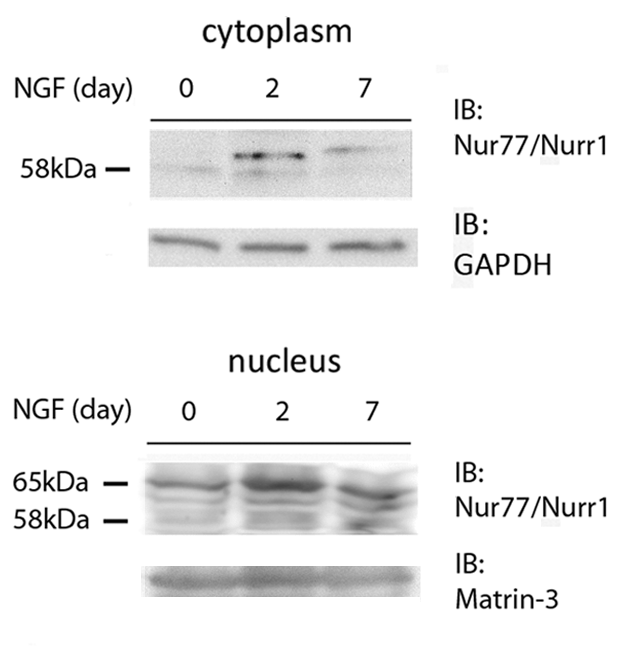

Supplement: Figure S4 — NGF-induced upregulation of Nur77/Nurr1 in PC12 cells. Western blotting revealed transient NGF-induced increases in Nur77/Nurr1 protein bands in the cytoplasm and in the nucleus. Pan Nur77/Nurr1 antibody (Santa Cruz) was used and the signals were normalized to GADPH (cytoplasmic) and matrin-3 (nuclear). (TIF) [file pone.0068931.s004.tif]

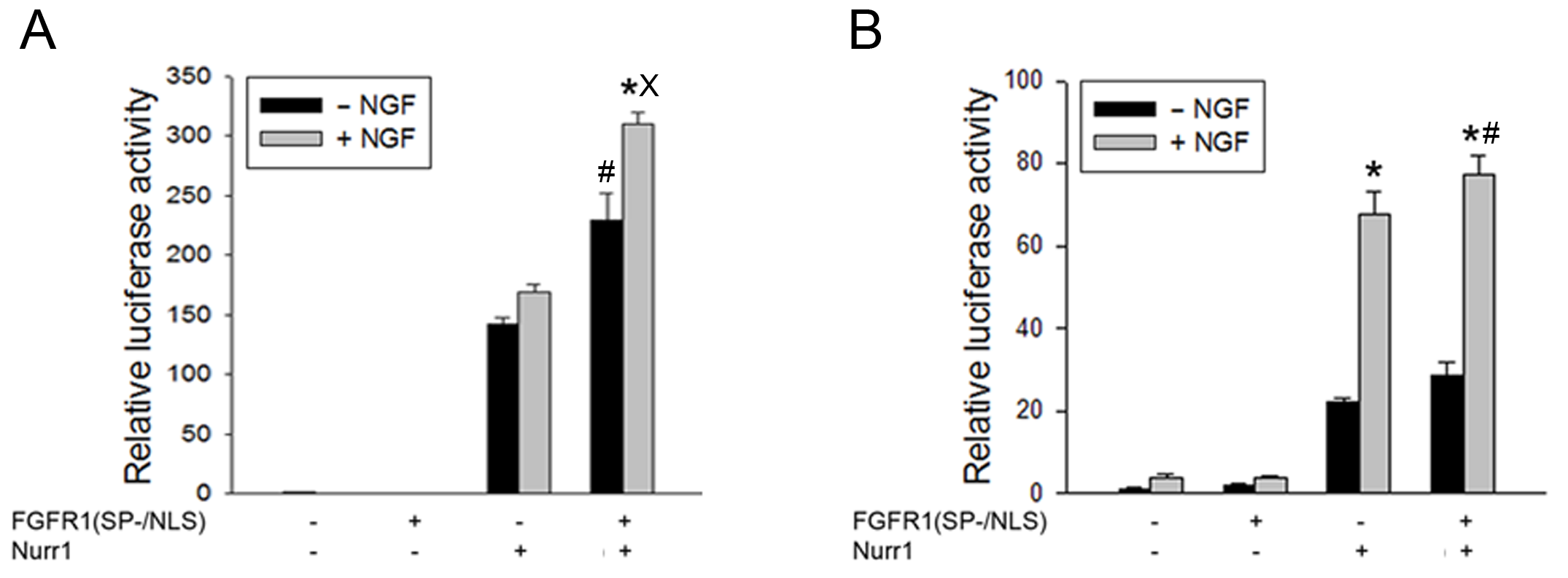

Supplement: Figure S5 — Nuclear FGFR1 and NGF augment Nurr1-mediated transcription. (A) PC12 cells were transfected with a NurRE-Luc reporter and Nurr1, FGFR1(SP−/NLS) or control β-galactosidase and treated with NGF or control medium for 6 h. FGFR1(SP−/NLS) has no significant effect on NurRE-Luc activation in the absence of Nurr1. However, the transcriptional activity of Nurr1 on the NurRE reporter is strongly enhanced by nuclear FGFR1(SP−/NLS) in both NGF treated and non-treated conditions (One-Way ANOVA *p<.001 different to no NGF treatment in individual groups; #p<.001 different to Nurr1 only; Xp<.001 different to Nurr1+ NGF). (B) PC-12 cells were transfected with a NBRE-Luc reporter and with Nurr1, FGFR1(SP−/NLS) or control β-galactosidase and treated with NGF or control medium for 24 h. No significant change between control and FGFR1(SP−/NLS) groups in the absent of Nurr1. The transcriptional activity of Nurr1 on the NBRE reporter is only slightly enhanced by nuclear FGFR1(SP−/NLS) after NGF treatment (One-Way ANOVA *p<.001 different to no NGF treatment in individual groups; #p<.05 different to Nurr1+ NGF). (TIF) [file pone.0068931.s005.tif]
