# Supplementary material for: NGF-Induced Cell Differentiation and Gene Activation Is Mediated by Integrative Nuclear FGFR1 Signaling (INFS)
Source: PLoS One. 2013 Jul 10;8(7):e68931. doi: 10.1371/journal.pone.0068931 (PMC3707895; doi:10.1371/journal.pone.0068931)
Supplement: Table S1 — Primers used for qPCR. (DOCX) [file pone.0068931.s006.docx]

| **Gene** | **Primers** | **Products (bp)** |
| --- | --- | --- |
| FGFR1 | \| 5’- GCTGCCAAGACGGTGAAATTC \| \| --- \| \| 5’- GTAGTTGCCCTTGTCAGAAGGC \| | 177 |
| FGF2 | \| 5’- GAACGGCGGCTTCTTCCTGCGC \| \| --- \| \| 5’- CACTCTTCTGTAACACACTTAG \| | 198 |
| TH | \| 5’- CGGACGGCGACAGAGTCTCATCG \| \| --- \| \| 5’- GAACAGCATTCCCATCCCTCTC \| | 143 |
| DCX | \| 5’- GCATTGCTGGCTGACCTGACTC \| \| --- \| \| 5’- GACCGACCAGTTGGGATTGAC \| | 201 |
| βIII-Tubulin | \| 5’- CAGAGTGGTGCTGGCAACAAC \| \| --- \| \| 5’- CGCTGAAGGTGTTCATGATGC \| | 226 |
| Nurr1 | \| 5’- GACAACTACAGCACAGGCTACG \| \| --- \| \| 5’- GATCGTCCCACATCGGGCTATG \| | 244 |
| Nur77 | \| 5’- CAGGTGTATGGCTGCTACCCTGGC \| \| --- \| \| 5’- CTTGGGCAACTGCTCAGTCCATAC \| | 219 |
| Cyclophilin A | \| 5’ - AACTTTCGTGCTCTGAGC \| \| --- \| \| 5’ - ATGGCGTGTGAAGTCACC \| | 124 |

**Table S1. Primers used for qPCR.**
