# Supplementary material for: NGF-Induced Cell Differentiation and Gene Activation Is Mediated by Integrative Nuclear FGFR1 Signaling (INFS)
Source: PLoS One. 2013 Jul 10;8(7):e68931. doi: 10.1371/journal.pone.0068931 (PMC3707895; doi:10.1371/journal.pone.0068931)
Supplement: Table S2 — Primers used for Chromatin Immunoprecipitation (ChIP). (DOCX) [file pone.0068931.s007.docx]

| **Gene** | **Site** | **Primers** | **Products (bp)** |
| --- | --- | --- | --- |
| TH | NBRE | \| 5’- AGCTCATAAGAGCTTTCAGATTATC \| \| --- \| \| 5’- CTGAGACAGGGTGGATCCCAG \| | 205 |
| TH | NurRE-like site | \| 5’- AGGTTATAGTTCTAACATGAG \| \| --- \| \| 5’- GCCTCCGTCCCATTAGATCTAATTG \| | 200 |
| DCX | NBRE | \| 5’-GCTCAGGACTTTGCATCCACCACTG \| \| --- \| \| 5’- TGGCCTGAATACATAGCAGTGG \| | 219 |
| DCX | NurRE-DR2 site | \| 5’- GAGGATCAATTAAGGAATAATAC \| \| --- \| \| 5’- ATGTTGCACAGTACATTCCTGTGAG \| | 253 |
| FGF2 | NBRE | \| 5’-TCTACCCTCACTCACAGTGGAGTG \| \| --- \| \| 5’- ACTCCAAGAACTGACTGAGTTG \| | 192 |
| FGF2 | NurRE-DR2 site | \| 5’- CTGAGAGAAGGTGTTCCGGGATC \| \| --- \| \| 5’- CATTAATATGTACAGCTCTTATAT \| | 210 |
| Nur77 | NurRE-like site | \| 5’- AGTCCTGTTGCAAGCTGTGCT \| \| --- \| \| 5’- TCAGGTGGCTCACAACCATCTG \| | 233 |
| Nurr1 | NurRE-like site | \| 5’- CATCCTGATTGAATAATCTTATCAT \| \| --- \| \| 5’- CATTTACCAGAAGAAAATTGATAGC \| | 184 |
| BIII-Tubulin | NBRE-like site | \| 5’- GTACTTGTCGGGTATTGTGCATC \| \| --- \| \| 5’- TGCTGAGTCGCTTGAGGTGGGAG \| | 204 |
| BIII-Tubulin | NurRE-like site | \| 5’- GATCTGTTTGGACTGTTCTTAGG \| \| --- \| \| 5’- TGTTGTTGTCATGTGGACATTAGC \| | 324 |
| Cyclophilin A |  | \| 5' - AACTTTCGTGCTCTGAGC \| \| --- \| \| 5' - ATGGCGTGTGAAGTCACC \| | 907 |

**Table S2. Primers used for Chromatin Immunoprecipitation (ChIP).**
